# Comparative genomics of nucleotide metabolism: a tour to the past of the three cellular domains of life

**DOI:** 10.1186/1471-2164-15-800

**Published:** 2014-09-17

**Authors:** Dagoberto Armenta-Medina, Lorenzo Segovia, Ernesto Perez-Rueda

**Affiliations:** Departamento de Ingeniería Celular y Biocatálisis, Instituto de Biotecnología, UNAM Av. Universidad 2001, Cuernavaca, Morelos, CP 62210 México; Unidad Multidisciplinaria de Docencia e Investigación, Sisal Facultad de Ciencias, UNAM, Sisal, Yucatán México

**Keywords:** Nucleotide metabolism, Comparative genomics, Evolution, Sequence profiles, Enzymes, LCA

## Abstract

**Background:**

Nucleotide metabolism is central to all biological systems, due to their essential role in genetic information and energy transfer, which in turn suggests its possible presence in the last common ancestor (LCA) of *Bacteria*, *Archaea* and *Eukarya*. In this context, elucidation of the contribution of the origin and diversification of *de novo* and salvage pathways of nucleotide metabolism will allow us to understand the links between the enzymatic steps associated with the LCA and the emergence of the first metabolic pathways.

**Results:**

In this work, the taxonomical distribution of the enzymes associated with nucleotide metabolism was evaluated in 1,606 complete genomes. 151 sequence profiles associated with 120 enzymatic reactions were used. The evaluation was based on profile comparisons, using RPS-Blast. Organisms were clustered based on their taxonomical classifications, in order to obtain a normalized measure of the taxonomical distribution of enzymes according to the average of presence/absence of enzymes per genus, which in turn was used for the second step, to calculate the average presence/absence of enzymes per Clade.

**Conclusion:**

From these analyses, it was suggested that divergence at the enzymatic level correlates with environmental changes and related modifications of the cell wall and membranes that took place during cell evolution. Specifically, the divergence of the 5-(carboxyamino) imidazole ribonucleotide mutase to phosphoribosylaminoimidazole carboxylase could be related to the emergence of multicellularity in eukaryotic cells. In addition, segments of salvage and *de novo* pathways were probably complementary in the LCA to the synthesis of purines and pyrimidines. We also suggest that a large portion of the pathway to inosine 5’-monophosphate (IMP) in purines could have been involved in thiamine synthesis or its derivatives in early stages of cellular evolution, correlating with the fact that these molecules may have played an active role in the protein-RNA world. The analysis presented here provides general observations concerning the adaptation of the enzymatic steps in the early stages of the emergence of life and the LCA.

**Electronic supplementary material:**

The online version of this article (doi:10.1186/1471-2164-15-800) contains supplementary material, which is available to authorized users.

## Background

Metabolism represents an intricate ensemble of enzyme-catalyzed reactions that lead to synthesis and degradation of compounds within the cell. In recent years, an increasing amount of information on metabolism from different species has become available, allowing for comparative genomic-scale studies on the evolution of specific pathways or whole metabolic networks [1–4]. Metabolism can be considered one of the most ancient biological networks, where nodes represent substrates or enzymes and edges represent the relationships between them. From this perspective, the study of metabolic networks is focused on describing topological properties, such as the existence of functional modules, giving special relevance to clustering and motif formation, and showing the existence of similar attributes to the small-world and scale-free networks [3].

Therefore, two main hypotheses on the origin and evolution of enzyme-driven metabolism have been postulated based on the notion that gene duplication, followed by divergence, can lead to the origin of new metabolic reactions. The “stepwise hypothesis” [5] suggests that, in the case where a substrate tends to be depleted, gene duplication can provide an enzyme capable of supplying the exhausted substrate, giving rise to homologous enzymes that catalyze consecutive reactions. On the other hand, the “patchwork hypothesis” [6] proposes that duplication of genes encoding promiscuous enzymes (capable of catalyzing multiple reactions) allows each descendant enzyme to specialize in one of the ancestral reactions.

In this regard, it is plausible that a small number of enzymes with broad specificity existed in early stages of metabolic evolution. Genes encoding these enzymes would have been duplicated, generating enzymes that, through sequence divergence, became more specialized [7].

Collectively, these studies have highlighted the contribution of gene duplication in the evolution of metabolism [4]. In recent works, the universal occurrence of some pathways and branches, such as diverse amino acid pathways, in modern species suggests that they existed in the last common ancestor (LCA) of *Bacteria*, *Archaea* and *Eukarya* [1, 8]. However, despite the importance of nucleotide metabolism in all organisms, few studies have addressed this issue by using genomic approaches [9, 10]. Nucleotide metabolism is central in all living systems, due to its role in transferring genetic information and energy. Indeed, it has been described as one of the ancient metabolisms in evolution. Specifically, the emergence of an ancestral folding or P-loop hydrolase appeared parallel to this metabolism [11], reinforcing its antiquity. In addition, many of the intermediates associated with this metabolic module have been intimately associated with prebiotic chemistry and the origin of life [10, 12, 13]. In this regard, we adopted a multigenomic strategy for the reconstruction and analysis of the metabolism of nucleotides, evaluating the contribution of the origin and diversification of *de novo* and salvage pathways for nucleotides in the evolution of organisms. In addition, these analyses allow the identification of a metabolic link between the LCA and the first steps in the structure of biological networks [14–16]. Our strategy reveals some general rules concerning the adaptation of the first predominant chemical reactions to enzymatic steps in the LCA and allows us to infer environmental issues in the early stages of the emergence of life. In addition, it was possible to determine the presence of *de novo* biosynthesis pathways of the ribonucleotides and deoxyribonucleotides of uracil and cytosine for pyrimidine metabolism associated with the LCA. Finally, we found differences in the enzymes for nucleotide metabolism that correlated with environmental changes and with associated cellular architecture adaptations; such is the case for the enzymes involved in the synthesis of 5-aminoimidazole ribonucleotide (AIR) to (4-carboxyaminoimidazole ribonucleotide) (CAIR). These findings suggest the presence of HCO_3_^-^ in primitive seas and its use as one of the main carbon sources for the first organisms. Our main results derived from the taxonomical distribution of enzymatic families belonging to nucleotide metabolism, supported by experimental evidence, are described in this report.

## Results and discussion

### Taxonomic distribution of nucleotide metabolic enzymes

The taxonomic distribution of proteins provides clues concerning the relative occurrence of the enzymes and their branches and paths for the evolution of metabolism [1, 8, 11, 17, 18]. In this regard, the origin and evolution of nucleotide metabolism was traced in organisms belonging to the three cellular domains, *Bacteria*, *Archaea* and *Eukarya*, by an exhaustive evaluation of the taxonomic distribution of their enzymatic repertoires. Therefore, each enzymatic activity encoded by an EC number can exhibit different profiles, which are individual vectors of the presence/absence of homologous enzymes in all the genomes. In total, 151 profiles associated with 120 enzymatic reactions related to nucleotide metabolism were used to scan 1,606 genomes by using RPS-BLAST. (Additional file 1: Figure S1 and Additional file 2: Figure S2 and Additional file 3: Tables S1 and Additional file 4: Table S2). Based on these comparisons, evolutionary origins of nucleotide metabolism can be traced, even close to the LCA of all organisms. In this regard, enzymes widely distributed across the three cellular domains are proposed to have been present in the LCA [8, 11, 18–20]. Alternatively, enzymes constrained to specific clades or cellular domains would suggest adaptations of organisms or cellular domains to specific lifestyles. Based on these considerations, we discuss our most notable results in the following sections.

### Evolution of purine metabolism

#### De novo purine biosynthesis

The *de novo* biosynthesis of purines, starting from d-ribose-1-phosphate to inosine 5’-monophosphate (IMP) production, the main intermediate in the synthesis of ribonucleotides and deoxyribonucleotides, guanine and adenine, follows a linear branch. The first step is associated with phosphoglucomutase (EC 5.4.2.2) or phosphopentomutase (5.4.2.7) and the second step is associated with ribose-phosphate diphosphokinase (2.7.6.1); both steps are necessary for the synthesis of 5-phospho-alpha-d-ribosy-1-pyrophosphate (PRPP), which starts from d-ribose-1-phosphate. Based on their taxonomical distribution, the enzymes associated with the 5.4.2.2 and 2.7.6.1 catalytic activities were identified as being widely distributed among *Bacteria*, *Archaea* and *Eukarya*, suggesting the probable existence of PRPP biosynthesis in the LCA. Indeed, PRPP is a key precursor for biosynthesis in the *de novo* and salvage pathways for purines and pyrimidines; however, this intermediary is unstable and susceptible to hydrolysis. Therefore, it is probable that its abiotic synthesis, if it occurred, was not enough to maintain the biosynthesis in the LCA [10].

Therefore, the first step for purine biosynthesis, the catalysis to ribose-5-phosphate starting from ribose 1-phosphate, is achieved by either of the two enzymes related to the enzymes EC 5.4.2.2 and 5.4.2.7. These two enzymes are analogous, since no homology at the sequence or structural level was detected. The enzyme 5.4.2.7 is partially distributed in *Bacteria*, mainly in free-living organisms associated with a host, such as *Streptococcus pneumoniae* and *Lactobacillus rhamnosus*; however, it was not found in archaeal and eukaryal organisms, suggesting that its emergence was posterior to the LCA divergence, probably as a secondary adaptation associated with the bacterial host.

**Figure 1:**
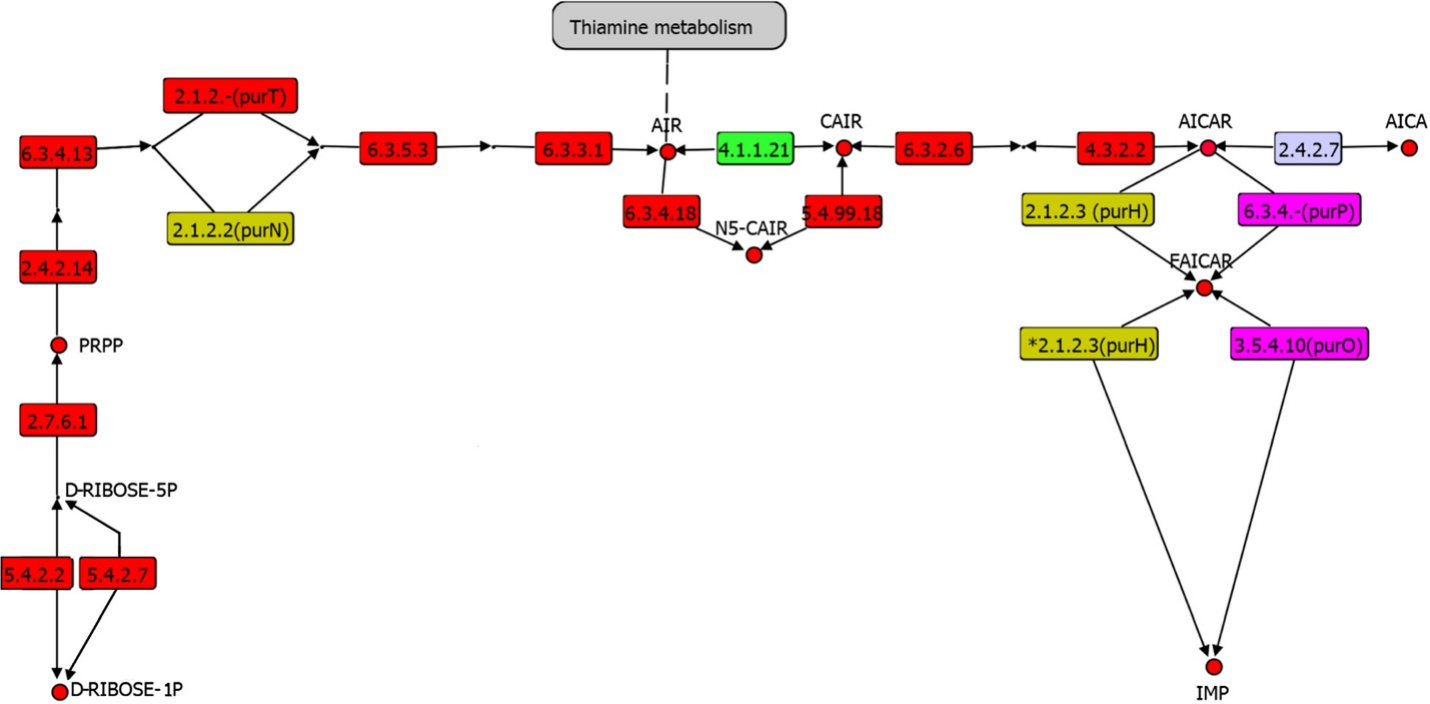
**Route of** ***de novo*** **biosynthesis towards IMP.** In red are the enzymatic steps associated with the LCA. In green is the enzymatic synthesis towards CAIR. In pink, are the enzymatic steps specifically identified in *Archaea* associated with the synthesis of AICAR to IMP. In gold are the folate-dependent enzymatic steps. The asterisk shows the second catalytic activity, IMP cyclohydrolase, achieved by PurH. The precursor PRPP is also indicated.

Starting from the intermediary PRPP, in the linear branch towards IMP biosynthesis, we identified enzymes belonging to five catalytic steps (amidophosphoribosyltransferase, 2.4.2.14; phosphoribosylamine-glycine ligase, 6.3.4.13; phosphoribosylglycinamide formyltransferase, 2.1.2.2; phosphoribosylformylglycinamidine synthase, 6.3.5.3; phosphoribosylformylglycinamidine cyclo-ligase, 6.3.3.1) required for the transformation of PRPP into AIR [21]. Most of these enzymes were identified as widely distributed in the three cellular domains, suggesting their presence in the LCA (Figure 1). The enzymatic step associated with EC 2.1.2.2 is responsible for the transformation of glycinamide ribotide (GAR) to formyglycinamide ribotide (FGAR) and could be carried out by two enzymes associated with different evolutionary families, PurN (Figure 1 Gold box) or phosphoribosylglycinamide formyltransferase and PurT or phosphoribosylglycinamide formyltransferase 2 [9]. Proteins associated with the PurN family use derivatives from folate synthesis as substrates. This family was identified as widely distributed in *Bacteria* and *Eukarya* and partially distributed in *Archaea*. Alternatively, proteins from the PurT family were partially distributed in *Archaea* and *Bacteria* and sparsely in *Eukarya*. It is probable that the PurT enzymatic family could have been present in the LCA, with posterior loss events in *Eukarya* due to its requirement for formate as a substrate. In this regard, formate is described as one-carbon donor and one of the main molecules present in prebiotics conditions, prior to folate metabolism [22–24]. In a posterior phase, the emergence of folate biosynthesis might have facilitated the emergence of PurN (Figure 1, gold box), thereby achieving the co-occurrence of PurN and PurT in the LCA. Indeed, previous works have suggested that the emergence of PurT preceded the emergence of PurN, mainly because PurT utilizes a more primitive substrate prior to the folate-dependent pathway [24]. One of the evolutionary pressures for the selection of PurN instead of PurT in eukaryotic organisms could be associated with the emergence of the mitochondrial respiratory chain. It has been shown that the PurT substrate, formate, is toxic and binds to cytochrome *c* oxidase-like [25, 26], uncoupling the redox reactions and favoring the selection of PurN in eukaryotic organisms.

Another enzymatic step identified in the purine biosynthetic pathway is achieved by the phosphoribosylformylglycinamidine synthase (6.3.5.3) that transforms FGAR to formylglycinamidine-ribonucleotide (FGAM). This enzyme is composed of two catalytic subunits, PurQ and PurL. In general, this enzyme may occur as a multidomain protein or as a multiprotein complex, where the subunits L and Q are encoded independently. Altogether, we identified a co-occurrence of these subunits in the three cellular domains. Finally, the last step towards AIR transformation is carried out by the cyclo-ligase phosphoribosylformylglycinamidine (EC 6.3.3.1), which transforms FGAM to AIR, and in turn this enzyme was found to be widely distributed in the three cellular domains, suggesting its presence in the LCA.

The product of the steps previously described, the AIR 5-amino-1-(5-phospho-d-ribosyl) imidazole-4-carboxylate, can be transformed by two pathways; in the first one, only one reaction, catalyzed by 4.1.1.21 (AIR carboxylase) (Figure 1, green box), is involved in CAIR synthesis; in the second, a two-step pathway, entails two reactions involving N5-carboxyaminoimidazole ribonucleotide synthase (6.3.4.18) and 5-(carboxyamino) imidazole ribonucleotide mutase (5.4.99.18). When the taxonomical distribution of these enzymes was evaluated, it was found that the N5-carboxyaminoimidazole ribonucleotide synthase (6.3.4.18) was widely distributed among all the organisms in the three cellular domains, whereas the enzyme 5-(carboxyamino) imidazole ribonucleotide mutase (5.4.99.18) exhibited a wide distribution in *Bacteria* and *Archaea* and in some eukaryal clades, suggesting their ancestry in all the organisms with posterior gene loss in *Eukarya* (Figure 1). Additionally, these enzymes were analyzed at the sequence level, where the 5-(carboxyamino) imidazole ribonucleotide mutase (5.4.99.18) was evolutionarily related to AIR carboxylase (4.1.1.21) [27]. Indeed, at the structural level both enzymes share a common evolutionary origin, belonging to the N5-CAIR mutase (phosphoribosylaminoimidazole carboxylase superfamily). The observation that AIR carboxylase (4.1.1.21) is almost exclusively distributed in the *Eukarya* suggests its posterior subfunctionalization from 5-(carboxyamino) imidazole ribonucleotide mutase (5.4.99.18) [28].

The discovery of the emergence of these branches is interesting, because it allows us to infer the processes associated with environmental changes on Earth. In this context, Tribunskikh *et al.* [29] suggested that the divergence of the phosphoribosylaminoimidazole carboxylase (4.1.1.21) to 5-(carboxyamino) imidazole ribonucleotide mutase (5.4.99.18) was a consequence of decreasing atmospheric CO_2_, which resulted in the addition of the 5-(carboxyamino) imidazole ribonucleotide synthase (6.3.4.18) and a change of specificity and, by consequence, in the two-step pathway. Those authors supported their proposal by the fact that the two-step pathways could work at low CO_2_ levels, under aerobic or anaerobic conditions. However, our data suggest an alternative scenario where, although the concentration of CO_2_ in the primitive atmosphere was high, acidic conditions in early oceans favored the formation of HCO_3_^-^. This is consistent with simulations showing that the concentration of HCO_3_^-^ oscillated between levels 30 to 30,000 times higher in the early oceans than current levels [30]. Interestingly, the enzyme 6.3.4.18, which is associated with the two-step pathway, uses HCO_3_^-^ as a substrate, and HCO_3_^-^ is considered a dominant form of CO_2_ in early oceans [30, 31], which together with our data of taxonomic distribution supports the notion that this pathway preceded the path where the enzymatic reaction catalyzed by 4.1.1.21 uses CO_2_ as the substrate. Indeed, it has been experimentally shown that in aqueous solutions with high concentrations of KHCO_3_, AIR is easily converted into CAIR in the absence of enzymes [29, 32, 33]. This mechanism could take place through the accumulation of the intermediate N-CAIR (N5-carboxy-AIR, or N5-carboxyaminoimidazole ribonucleotide), which then undergoes a rearrangement to CAIR. These reactions appear to be the template whereby the enzymatic activities adapted in the two-step pathway, widespread in the cellular domains and probably in the LCA. Probably, HCO_3_^-^ levels had a steady decline due to the reduction of atmospheric CO_2_, as documented in the evolution of the terrestrial atmosphere [31]. This reduction could have selected the enzymes 6.3.4.18 and 5.4.99.18, which transform AIR to N-CAIR and N-CAIR to CAIR, respectively.

Subsequently, once the atmosphere was provided with oxygen, the emergence of mitochondria and eukaryotic cells was possible. In this regard, HCO_3_^-^ is one of the main products of mitochondrial respiration, which follows a pH-dependent conversion to CO_2_, converting an impermeable anion into a gas that can diffuse through membranes [34]. The transformation of HCO_3_^-^ accumulated in cells with mitochondrial activity towards CO_2_ may have resulted in a more efficient regulation of intracellular pH in eukaryotic cells, and in parallel, to the use of CO_2_ as a carbon source. All these processes might have favored multicellularity, because cells with nutrients availability and high mitochondrial activity as the consequence of oxidative respiration could provide a permeable carbon source to other cells with low nutrients availability. Altogether, the taxonomic distribution data, chemical synthesis information, and primitive ocean simulations data, support the divergence of the enzyme 5.4.99.18 to 4.1.1.21 by selecting a CO_2_ binding site. Although the selection of a CO_2_ binding site in metabolism is not exclusive to this protein family, the selection of a CO_2_ binding site in these enzymes could favor the development of multicellularity in eukaryotic cells.

The last two steps in the synthesis of IMP correspond to the transformation of 5-aminoimidazole-4-carboxamide ribonucleotide (AICAR) and 5-formamidoimidazole-4-carboxamide ribotide (FAICAR). In the first step (2.1.2.3), AICAR formyltransferase activity is required, whereas for the second step (3.5.4.10), IMP cyclohydrolase activity is required. The gene for the bifunctional folate-dependent enzyme PurH, which is widely distributed in *Bacteria* and *Eukarya* and partially in *Archaea*, encodes both activities [35]. PurH exhibits two independent catalytic sites, in which each half of the enzyme catalyzes an independent reaction. The transformation to IMP by the bifunctional enzyme PurH may have been posterior to the emergence of the folate synthesis pathway (Figure 1, gold box). In addition, both catalytic reactions performed by PurH could have emerged in *Archaea* to be carried out by different proteins that achieve each step independently. In this regard, the first reaction is carried out by the formate-dependent PurP enzyme, whereas the second one is achieved by the IMP cyclohydrolase PurO (Figure 1, magenta). In both cases, the catalytic mechanism is very similar to that of its counterpart PurH, and they are exclusively distributed in *Archaea*; however, these two enzymes do not maintain relationships at the sequence or structural level with PurH [9, 35]. In summary, all these results suggest that PurP and PurO emerged posterior to archaeal divergence.

Alternatively, the archaeal cenancestor may have brought the formate (PurT) and folate-dependent (PurN-PurH) families, as seen in *Halobacteria*. The emergence of PurP in *Archaea* was probably related to the availability of formate in the environment, which agrees with the fact that most of the *Archaea* clades are methylotrophs. In turn, PurP may have duplicated from families associated with the LCA [carbamoyl-phosphate synthase, 6.3.5.5; phosphoribosylamine-glycine ligase, 6.3.4.13; 5-(carboxyamino) imidazole ribonucleotide synthase, 6.3.4.18; phosphoribosylglycinamide formyltransferase, 2.1.2.2] belonging to nucleotide metabolism, partially replacing PurH in some archaeal clades. For instance, PurH was identified in some archaeal clades, such as *Halobacteria*, *Thermoplasmata*, and *Methanomicrobia* among others, including co-occurrence with PurP, suggesting that their activities are complementary in these organisms.

In the case of PurO, we did not find homology relationships with any other enzymatic family associated with nucleotide biosynthesis. In addition, we found a correlation between the presence of PurP and PurO; however, in *Archaea* some clades do not exhibit either PurH or PurO, which suggests that alternative enzymes performing the PurO function are probably present and remain to be described.

### Purine salvage pathway

#### Ribonucleotide adenine salvage pathway

**Figure 2:**
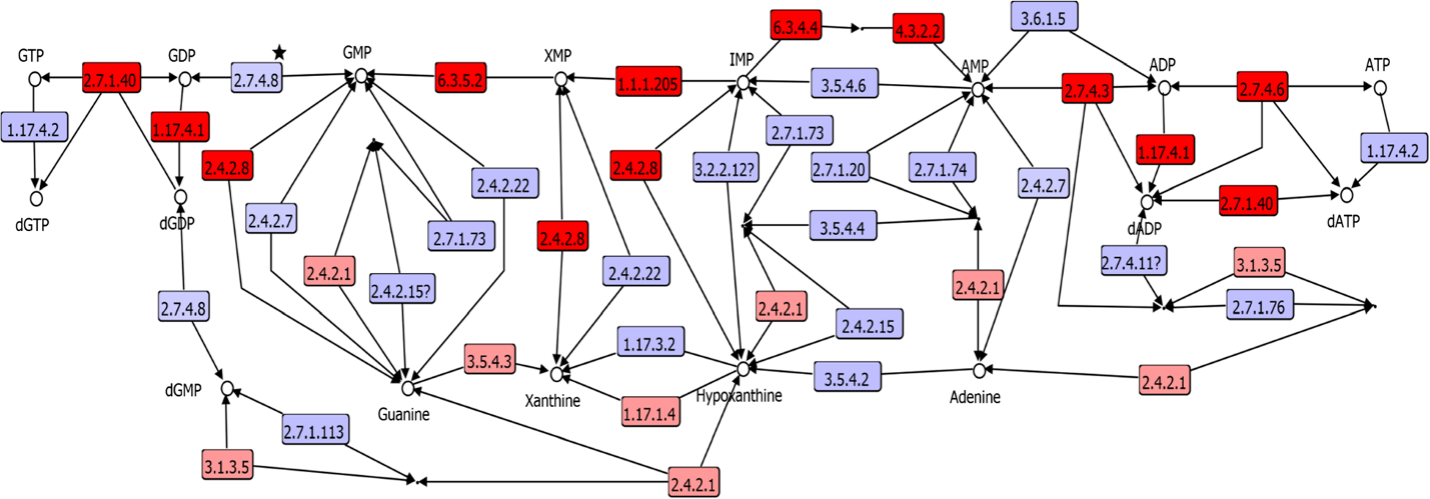
**Salvage routes of nucleotide and** ***de novo*** **biosynthesis starting from IMP.** In red are the enzymatic steps associated with the LCA; in pink, enzymatic steps with dubious taxonomical distribution pattern to be associated to the LCA are shown; in blue, enzymatic steps not associated to the LCA. The enzymatic step associated with the guanylate kinase family is indicated with a star.

In the purine salvage pathway, diverse pathways to generate adenine and guanine ribonucleotides and deoxynucleotides were identified. In this regard, the first pathway starts from hypoxanthine, via hypoxanthine-guanine phosphoribosyltransferase (PRTase) (2.4.2.8) (Figure 2), an enzyme with broad specificity and that is universally distributed in the three cellular domains. This enzyme achieves the biochemical transformation to IMP, starting from hypoxanthine and PRPP, for subsequent transformation to the ribonucleotide of adenine through the *de novo* adenyl-succinate pathway, which includes enzymes widely distributed in the three cellular domains (6.3.4.4, adenylosuccinate synthetase; 4.3.2.2, adenylosuccinate lyase; 2.7.4.3, adenylate kinase; 2.7.4.6, nucleoside diphosphate kinase; 2.7.1.40, pyruvate kinase), suggesting its presence in the LCA (Figure 2). For the transformation of alpha-deoxyribonucleotide, there are two routes, the pathway associated with the ribonucleoside-diphosphate reductase beta-subunit (small subunit) (1.17.4.1), the ribonucleoside-diphosphate reductase alpha-subunit (large chain family), and the 1.17.4.2 pathway, which contains adenosylcobalamin-dependent ribonucleoside-triphosphate reductase, and the anaerobic ribonucleoside-triphosphate reductase complex (NrdD and NrdG). The first of these pathways, for the formation of deoxyribonucleotides, is widely distributed in the three cellular domains, suggesting its presence in the LCA. Finally, the enzymes of the 1.17.4.2 pathway are partially distributed in *Bacteria* and *Archaea* but not in *Eukarya,* which makes it difficult to determine its presence in the LCA.

Alternatively, it is also possible to synthesize adenine deoxynucleotides and ribonucleotides. This pathway conserves the last three catalytic steps (2.7.4.3, 2.7.4.6 and 2.7.1.40) from the pathway previously described, *i.e.*, only the first step has been added, which is catalyzed by adenine phosphoribosyltransferase (a PRTase) (2.4.2.7) (Figure 2), an enzyme widely distributed in *Bacteria* and *Eukarya* and that starts from the adenine salvage pathway.

This enzyme may be specialized for the adenine substrate, posterior to the divergence of the LCA, from a broad-specificity ancestor, similar to the HPRTases (2.4.2.8).

#### Route of salvage of guanine ribonucleotides

Starting from the first salvaging route, previously described, which starts with hypoxanthine-guanine phosphoribosyltransferase (2.4.2.8), it is also possible to synthesize guanine ribonucleotide, adding to the enzymatic families widely distributed in the three cellular domains: inosine-5’-monophosphate dehydrogenase 1 (1.1.1.205) and GMP synthase (glutamine-hydrolyzing) subunit A and GMP synthase (glutamine-hydrolyzing) subunit B (6.3.5.2) (Figure 2). Because the hypoxanthine-guanine phosphoribosyltransferase (2.4.2.8) also exhibits specificity for guanine, it is possible to synthesize (ribonucleotide monophosphate guanine) guanosine 5’-monophosphate (GMP) in one step, starting from the guanine salvage pathway with PRPP; however, although the subsequent step with 2.7.4.8, performed by the guanylate kinase family, for transforming GMP to GDP is widely distributed in *Eukarya* and *Bacteria*, in *Archaea* only the reaction but not the enzyme has been identified (Figure 2, star) [36]. These data limit the possibility of making genomic and evolutionary comparisons between *Bacteria* and *Eukarya* to guarantee the possible presence of guanine deoxyribonucleotides in the LCA. However, subsequent steps for the synthesis of GDP to GTP are carried out with the nucleoside diphosphate kinase (2.7.4.6) and pyruvate kinase (2.7.1.40); both of these enzymes are widely distributed in the three cellular domains, and the step of GTP conversion to dGTP for the widely distributed enzyme ribonucleoside diphosphate reductase (1.17.4.1) suggests the presence of GTP and dGTP in the LCA.

**Figure 3:**
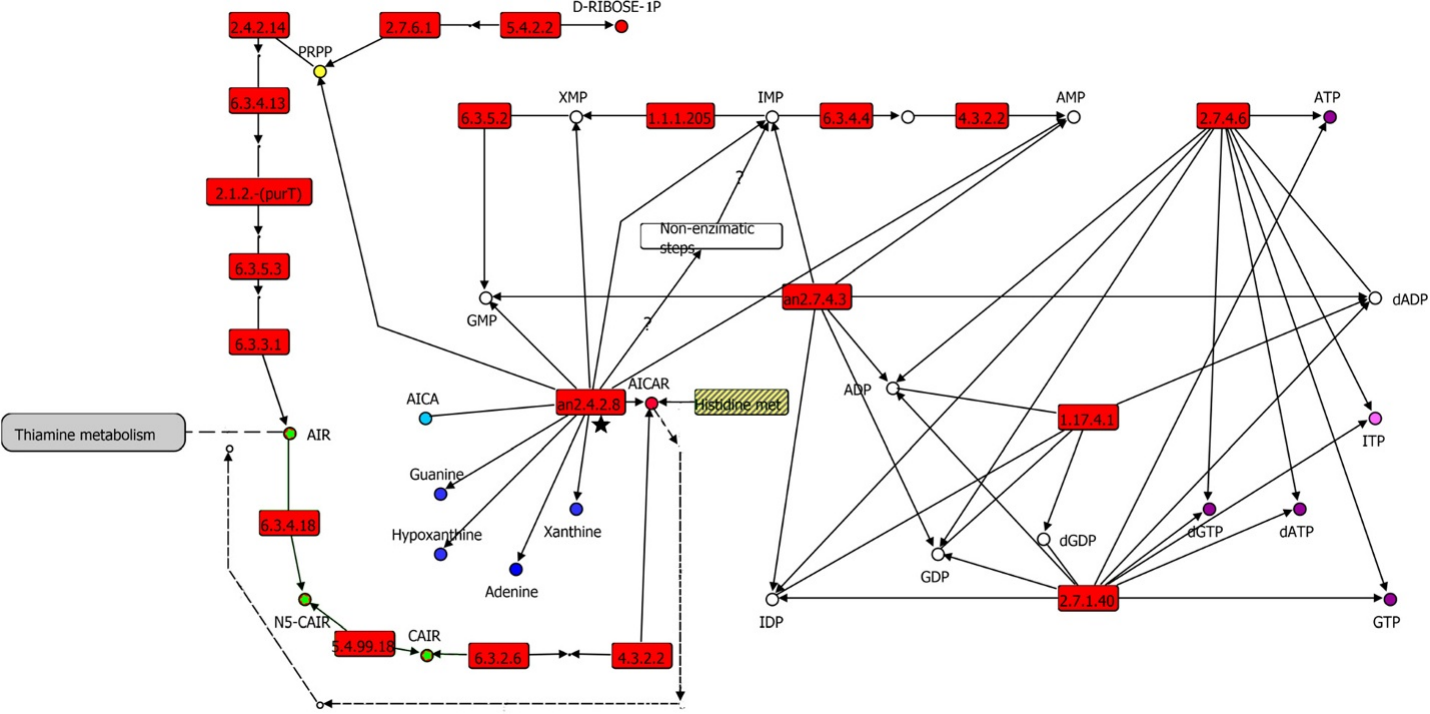
**Purine biosynthetic pathway associated with the LCA.** In red are all the steps associated with the LCA. In white is the semienzymatic pathway associated with an early stage of cell development, previously proposed by Becerra *et al.*[10]. The proposed ancestral histidine biosynthesis that feeds AICAR is displayed in a box with bars. The substrates with prebiotic origins are shown in blue circles. Ribonucleotides that may have occurred in the LCA are in purple circles. The thiamine (vitamin B_1_) metabolism is in gray. In dashed lines, is indicated the AICAR in backflow as a seed substrate to AIR.

Therefore, although the gene sequence associated with the guanylate kinase function in *Archaea* has not yet been identified, we suggest that this protein could exhibit a common origin with its counterpart in *Bacteria* and *Eukarya*, because most of the nucleotide kinases belong to the P-loop-containing nucleoside triphosphate hydrolases family. This family shows a broad specificity in recognition of ribonucleotides and deoxyribonucleotides [37]. In this regard, it has been reported that only two mutations are enough to introduce the adenylate kinase activity into guanylate kinase [38], suggesting a masking of guanylate kinase assignment by adenylate kinase. In addition, it is possible that an enzyme with broad specificity similar to adenylate kinase (Figure 3, star) recognizes NMP and converts it into NDP; thus, its specialization toward the guanylate kinase function occurred posterior to the divergence of the three cellular domains.

At present, members of the adenylate kinase family and in particular from the AK6 subfamily have been described as proteins that exhibit the ability to transform different NMPs to their corresponding NDPs, specifically, the AK6 subfamily transforms AMP, dAMP, CMP, dCMP, IMP, and GMP to their corresponding NDPs and dNDPs [39, 40]. This finding also suggests that an ancestral enzyme with broad specificity for the synthesis of ribonucleotides and deoxynucleotides could have been utilized in the LCA. It is also interesting that this enzyme could have used IMP as a substrate, since this nucleotide could have had an active role in the evolution of nucleic acids and the genetic code [41]. Interestingly, our results show that *Archaea* contain an adenylate kinase from the AK6-like subfamily, similar to *Eukarya* and *Bacteria*, suggesting that the function of guanylate kinase in archeal organisms, which has not yet been identified, could be carried out at least in part by enzymes of this subfamily.

#### Integrating the evolutionary analysis of the salvage and de novo purine pathways

The taxonomic distribution of enzymes associated with purine metabolism shows that segments of the *de novo* and salvage pathways were complementary and critical to the availability of nucleic acids before the divergence of the three cellular domains. This finding correlates with the chemical synthesis under prebiotic conditions for nitrogenous base precursors for the purine salvage pathway, as previously described [10, 13, 42, 43] (Figure 3, blue circles). The findings of those previous studies agree with the fact that these routes were dependent on PRPP biosynthesis, since it has been argued that PRPP, nucleosides, and nucleotides are susceptible to hydrolysis and thus are very unlikely prebiotic compounds. In addition, our data show a widely taxonomic distribution in the three cellular domains of the enzymes associated with the last two enzymatic steps (5.4.2.2 and 2.7.6.1) required for the transformation of ribose-1-phosphate to PRPP (Figure 3, yellow circle), supporting the biosynthesis of this molecule in the LCA.

Despite the possible absence of folate and folate-dependent enzymes PurH and PurN in early stages of life emergence (Figure 1, gold boxes), most of the enzymes associated with the *de novo* biosynthesis pathway for IMP may have occurred in the LCA (Figure 1), based on their wide taxonomical distribution in the three cellular domains. Posterior to early stages of life emergence and folate biosynthesis, the appearance of PurH could have completed the purine biosynthesis pathway, filling the gap between AICAR and IMP (Figure 1, PurH is shown in the gold boxes). In addition, the fact that PurP and PurO have been identified exclusively in *Archaea*, without homology with PurH, suggests that these proteins were generated posterior to the divergence of *Archaea* (Figure 1, magenta boxes).

Previous to the emergence of folate synthesis and by consequence of PurH, it is possible that in addition to the salvage routes, semienzymatic alternative routes, starting from the substrates 5-amino-4-imidazole carboxyamide AICA and PRPP, are transformed to AICAR by an ancestral phosphoribosyltransferase (PRTase), with subsequent nonenzymatic transformations to IMP, as has been previously proposed [10] (Figure 3, white boxes). Specifically, the adenine phosphoribosyltransferase (PRTases) (2.4.2.7) synthesize adenine ribonucleotides from the adenine salvage pathway and also transform AICA to AICAR. This enzyme may have become specialized posterior to the divergence of the LCA, from a broad-specificity ancestor, similar to the HGPRTase (PRTases) (2.4.2.8); indeed, this enzyme was found to be universally distributed among the three cellular domains and exhibits a similar catalytic mechanism. In evolutionary terms, the enzymes 2.4.2.7 and 2.4.2.8 belong to the phosphoribosyltransferases superfamily, suggesting a common evolutionary origin.

Based on data previously described, we suggest that an ancestral PRTase enzyme similar to the hypoxanthine-guanine phosphoribosyltransferase (2.4.2.8) (Figure 3, star) with broader specificity to diverse structurally related substrates, such as guanine, xanthine, hypoxanthine, adenine, and AICA, was present in the early stage of the emergence of life. This ancestral enzyme with prebiotic origins could have been involved in the transformation of AICA (Figure 3, light blue circle) [10] to AICAR, not only to feed semienzymatic IMP synthesis, as previously proposed [10] (Figure 3, white box), but also to a greater extent to thiamine (vitamin B_1_) biosynthesis. From this, it is interesting two pathways feed that thiamine metabolism as a bifurcation of the *de novo* IMP pathway and also with AICA in backflow as a seed substrate (Figure 3, following the direction of the dashed arrows). Additionally, the thiamine pathway may have been also fed from a third source by means of histidine metabolism (Figure 3, box with bars); the connection to purine biosynthesis results from an enzymatic step catalyzed by imidazole glycerol phosphate (IGP) synthase, which transforms *N*-(5-phosphoribosyl)-formimino-5-aminoimidazol-4-carboxamide ribonucleotide (PRFAR) into AICAR, which is then recycled into the *de novo* purine biosynthetic pathway, and imidazole-glycerol 3-phosphate, which leads to histidine. Interestingly, previous works have also suggested that the histidine biosynthetic route is ancient and related to the emergence of life [18]. Our proposal of an ancestral branch related to thiamine synthesis is consistent with the catalytic functions that have been proposed for this molecule in the early evolution of life, as suggested by its essential catalytic role in most of organisms and its requirement at several central points of anabolic and catabolic intermediary metabolism [44], as well as in semienzymatic pathways that may precede the actuals [45]. It is interesting this branch in the early emergence of life and before the constitution of the LCA could have been fed the thiamine metabolism in a semienzymatic way, as the AICAR-to-AIR transformation could occur in a facile, nonenzymatic chemical synthesis pathway [46].

In this regard, molecules of thiamine or its derivatives bind to the mRNA in the absence of cofactors or proteins in the three domains of life, forming a complex that sequesters the ribosome binding site and suggesting the existence of an ancestral form of riboswitches, which have been implicated in regulatory mechanisms [47]. Additionally, thiamine could have interacted with the RNA, leading to catalytically versatile ribozymes related to the RNA world, due to its catalytic and RNA binding capabilities [44].

Finally, we suggest that the guanylate kinase function could have been carried out in the LCA by an ancestral enzyme with broad specificity to nucleoside monophosphates (NMPs), similar to those in the AK6 adenylate kinase subfamily. Its specialization to guanylate kinase could have been posterior to archaeal divergence. In parallel, this ancestral enzyme may have provided the nucleotide inosine (Figure 3, pink circle), which has been suggested to play an active role in a rudimentary stage of the genetic code [41]. The ITP can compete with or replace the ATP and GTP binding sites in diverse proteins, such as RNA polymerase [48, 49], that may have maintained their affinity and specificity for ITP as a remnant of its ancestral role.

In addition, inosine maintains a strong structural similarity to guanine [50], and it may even have the same function for guanine pairing to cytosine in the codons. Currently, inosine has been found in the third position of anticodons, pairing with codons at bases U, C or A and thereby decreasing the need for the 61 tRNAs for each codon. In this regard, it has been suggested that inosine may have been produced by adenosine deamination or even in RNA-mediated catalysis through an early stage of emergence of the genetic code, and it was excluded in nucleic acids when the canonical Watson-Crick pairing evolved to avoid ambiguous rules in replication [41]. Posterior to the divergence of *Archaea* from the LCA, the specialization and divergence to guanylate kinase of members with broad specificity of the ancestral AK6 subfamily type could have increased the availability of guanine, favoring the replacement of inosine for guanine.

### Evolution of pyrimidine metabolism

#### De novo pyrimidine biosynthesis

**Figure 4:**
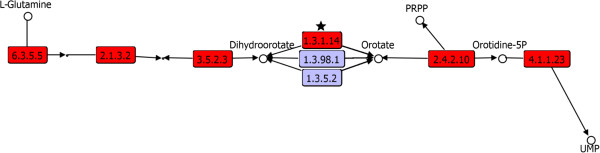
**The** ***de novo*** **pyrimidine biosynthesis pathway towards UMP.** The enzymes associated with the LCA are shown in red boxes. The enzymatic step associated with the proposed ancestral dihydroorotate dehydrogenase family is denoted with a star.

Based on taxonomical distributions, we evaluated the enzymes associated with *de novo* pyrimidine biosynthesis: carbamoyl-phosphate synthase large chain, carbamoyl-phosphate synthase small chain, 6.3.5.5; aspartate carbamoyltransferase 1, 2.1.3.2; dihydroorotase, DHOase family, 3.5.2.3; dihydroorotate dehydrogenase B (NAD^+^), catalytic subunit, dihydroorotate oxidase B, electron transfer subunit, 1.3.1.14; dihydroorotate dehydrogenase (quinone), 1.3.5.2; dihydroorotate dehydrogenase A (fumarate), 1.3.98.1; orotate phosphoribosyltransferase 1, PyrE1, 2.4.2.10; orotidine 5’-phosphate decarboxylase, 4.1.1.23; cytidylate kinase, 2.7.4.14; nucleoside diphosphate kinase, 2.7.4.6 (Figure 4). These enzymes form the branch of UTP *de novo* biosynthesis starting from l-glutamine. This entire branch may have occurred in the universal ancestor, based on the wide taxonomical distribution of the enzymes that compose it (Figure 4, red boxes). One of the most interesting steps of this pathway is the conversion of dihydroorotate to orotate, which is carried out by enzymes classified into two families of dihydroorotate group dehydrogenases (DHODs), according to the terminal electron acceptor and relationships at the sequence level [51]. In this regard, family 1 uses soluble electron acceptors. This family is widely distributed in gram-positive *Bacteria*, *Archaea* and in some unicellular eukaryotic organisms. In turn, this family is subdivided into DHODA (1.3.98.1) and DHODB (1.3.1.14), which are homodimeric and heterotetrameric, respectively. DHODA uses fumarate as the electron acceptor, whereas DHODB uses NAD^+^ [52]. Members of family 2 (1.3.5.2) are linked to the cell membrane and use quinones from the respiratory chain as electron acceptors [53]. These enzymes are mainly found in most eukaryotic organisms and gram-negative bacteria, in agreement with our taxonomical distribution results.

**Figure 5:**
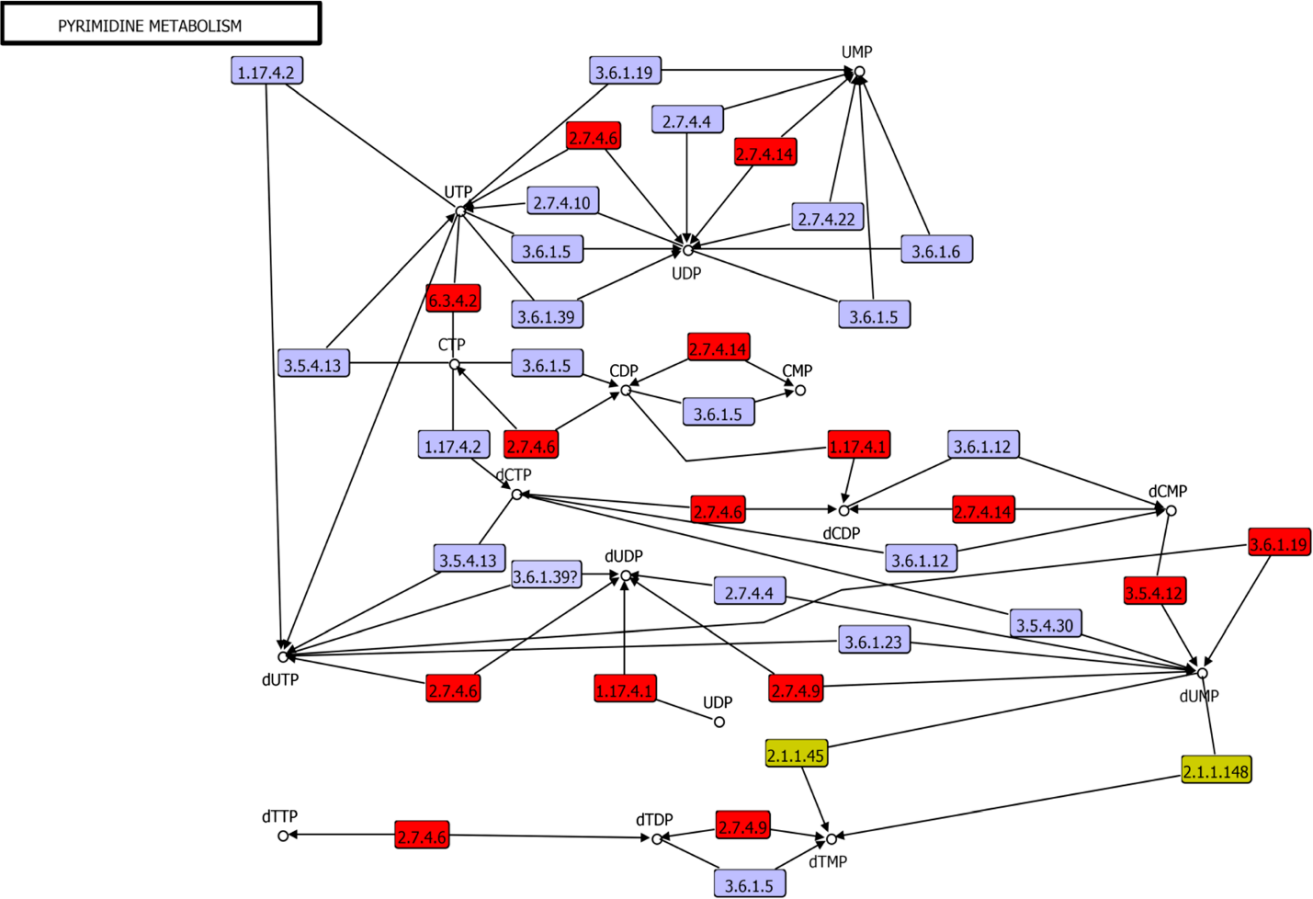
**Pyrimidine metabolism starting from UMP.** The enzymatic steps associated with the LCA are shown in red boxes. The folate-dependent enzymes for thymine biosynthesis are indicated in gold boxes. In blue boxes, are indicated enzymatic steps not present in the LCA.

In spite of these differences, both DHODs belong to the FMN-linked oxidoreductases superfamily, suggesting a common ancestor. Probably, this ancestral enzyme was similar in functional terms to members of the family 2 (Figure 4, star), according to its electron acceptor molecules, which have been previously described as one of the most abundant in extraterrestrial environments, and also that could have been delivered to the Earth around 4 billons years ago, making possible the prebiotic conditions needed for the emergence of life. Indeed, quinones have been found in meteorites in considerable amounts and also have been synthesized in a cloud chamber simulation with good yields [54, 55]. Additionally, quinones are spontaneously partitioned into model membrane systems, representing an evolutionary advantage to early organisms by providing some protection against UV radiation in the early Earth environment [56], and were posteriorly exploited for their capacity to pump protons across membrane bilayers [57]. In a posterior step, changes associated with the cell membrane and cell wall could lead to divergence of the DHODs from family 2 to family 1, via incorporation of soluble electron acceptors. Surprisingly, our proposal for DHOD family divergence is consistent with previous reports describing a transition from gram-negative to gram-positive bacteria [58, 59] and to drastic changes associated with the chemical constituents of the cell membrane in *Archaea*, such as glycerol stereochemistry [60], posterior to the divergence of the LCA. Furthermore, subdivision of family 1 might have occurred in the direction of DHODB to DHODA, since most of these enzymes have been found in gram-positive bacteria that have adapted to parasitic or symbiotic relationships, as shown by our taxonomical distribution results. The DHODA uses fumarate as a terminal electron acceptor, which in turn is used instead of oxygen as a terminal electron acceptor for succinate production, one of the essential processes controlling redox homeostasis for many parasites living under anaerobic conditions [53].A wide taxonomic distribution of the enzyme CTP synthase (6.3.4.2), which converts UTP to CTP, was also found in the three cellular domains, in addition to the enzyme for the subsequent steps, nucleoside diphosphate kinase (2.7.4.6), thioredoxin reductase NTRA (1.8.1.9) and ribonucleotide reductase (1.17.4.1), giving rise to deoxy-CTP. Based on the distributions of these enzymes, the presence of UTP, CTP and deoxy-CTP in the LCA is suggested (Figure 5, red boxes).

Concerning the transformation to deoxyuridine, two main routes have been described. One pathway starts from deoxy-CDP, whereas the second one starts from deoxy-CMP and which can be extended to transforming deoxyuridine. The route of deoxy-CDP requires two enzymatic steps: the first one requires the nucleoside diphosphate kinase (2.7.4.6), which produces dUTP, which is subsequently transformed by the inosine triphosphate pyrophosphatase 1 (nucleoside-triphosphate pyrophosphatase 1; 3.6.1.19) to dUMP. These two enzymes were identified as universally distributed in the three cellular domains; therefore, they can be associated with the LCA (Figure 5, red boxes). The second pathway converts dCMP to dUMP via cytidine/deoxycytidylate deaminase, which is associated with the catalytic activity of 3.5.4.12, whose members are partially distributed among the three cellular domains. Finally, a third pathway, which starts from dCTP and is catalyzed by deoxycytidine triphosphate deaminase (3.5.4.13), is absent in eukaryotes and partially distributed in *Bacteria* and *Archaea*. The assignment of the second and third pathways toward deoxyuridine transformation was within the LCA, based on the complex evolutionary history of their enzymes.

Finally, the transformation of deoxy-UMP to deoxy-TMP can be carried out through two folate-dependent enzymes, thymidylate synthase ThyX (2.1.1.148) and thymidylate synthase ThyA (2.1.1.45) [9] (Figure 5 in gold boxes). These enzymes are not homologous, suggesting an independent evolutionary origin. Both enzymes promote methylation by using the 5,10-methylenetetrahydrofolate (CH_2_-H_4_ folate) as a carbon donor. ThyA also uses CH_2_-H_4_ folate to produce dihydrofolate (H_2_-folate). In contrast, ThyX uses flavin adenine dinucleotide (FAD) and NAD (P) H as cofactors to form reduced tetrahydrofolate (H_4_-folate) [61]. ThyX is partially distributed in *Bacteria* and *Archaea* but is absent in eukaryotes; its counterpart, ThyA, is partially distributed in *Bacteria*, sparsely distributed in *Archaea* and widely distributed in *Eukarya*. In this context, it has been suggested that the catalytic differences between ThyA and ThyX influenced the evolution of bacterial genomes [61, 62]. In this regard, ThyA is 10 times more catalytically efficient than ThyX, and previous studies in more than 400 prokaryotic genomes have revealed that the catalytic capacity associated with ThyA correlates with its presence in organisms with large genome sizes [61, 62]. Our results related to its taxonomic distribution are consistent with those of earlier studies, which showed a pattern of anticorrelation for the presence/absence of these two enzyme families through bacterial clades, which has also been described as a mutual event of replacements between these two families [63], indicating that bacterial metabolism has modulated the size and composition of the bacterial genomes. Additionally, our data show the complete absence of ThyX and absolute presence of ThyA in eukaryotic genomes, suggesting that the influence of ThyA was a determinant in the eukaryotic genome size and in those organisms’ evolutionary potentials. In this context, it is difficult to determine whether some of these families were present in the LCA, based on their observed complex taxonomical distribution.

#### Pyrimidine salvage routes

In association with the salvage pathway for uracil ribonucleotide are two main routes that start from uracil. The first pathway comprises uridine kinase (2.7.1.48) and uridine phosphorylase (2.4.2.3). Uridine phosphorylase was found sparsely distributed in the three cellular domains and uses preformed uracil as a substrate. Uridine kinase is widely distributed in *Bacteria* and *Eukarya* and sparsely distributed in *Archaea*. Therefore, based on the taxonomical distribution patterns, it is difficult to determine whether all these enzymes were present in the LCA, due to their low distribution in *Archaea* genomes, which may also be a reflection of horizontal transfer events.

**Figure 6:**
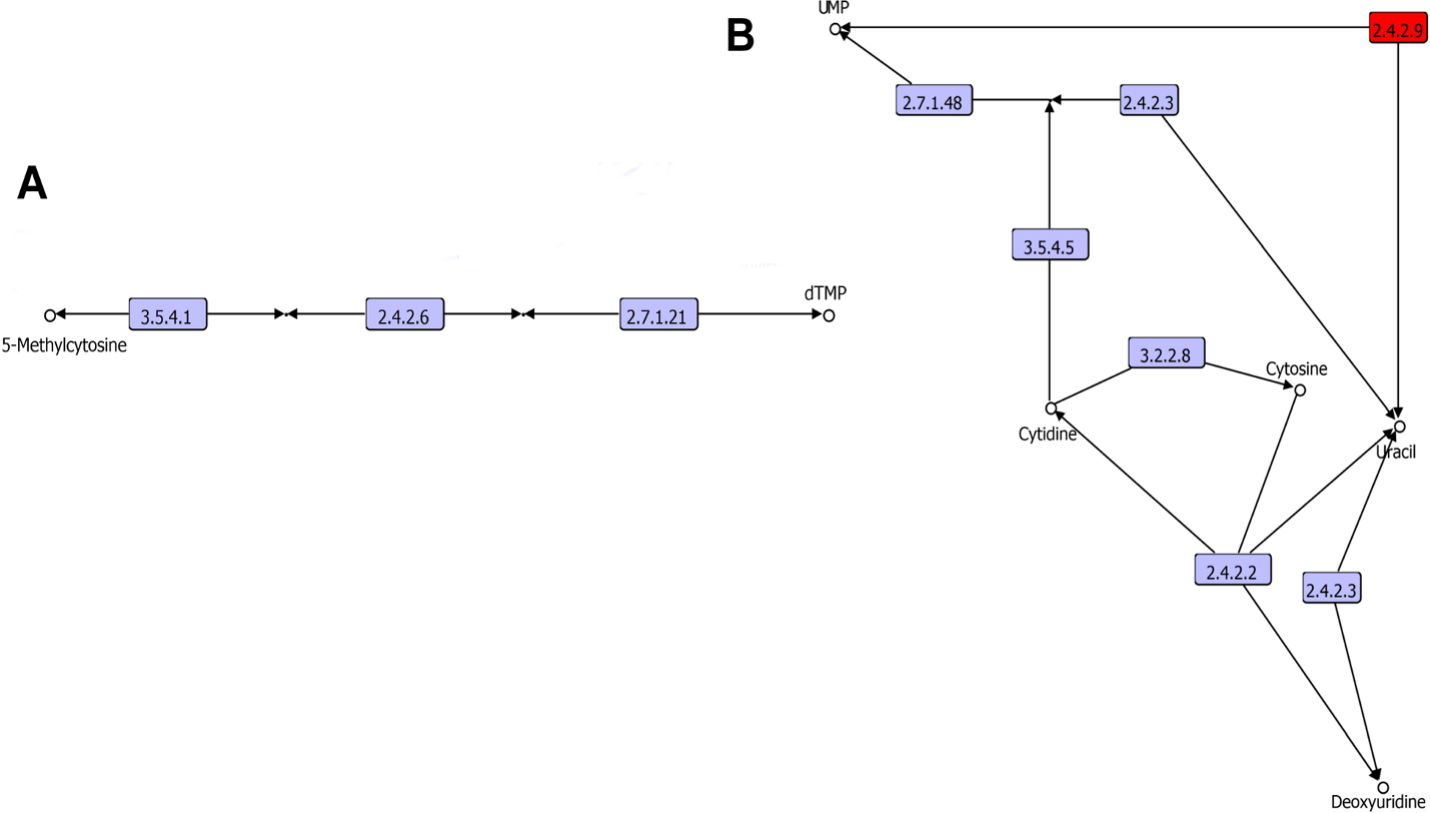
**Pyrimidine salvage routes. A)** The salvage thymine route. **B)** The salvage pathway for uracil and cytosine. The enzyme associated with the LCA is shown in red.

The enzymatic family of uridine phosphorylases uses uridine as a substrate, which can be provided by another pathway branch associated with cytidine deaminase (3.5.4.5) and pyrimidine-nucleoside phosphorylase (2.4.2.2). This branch uses cytosine as the starting substrate. Cytidine deaminase (3.5.4.5) maintains a similar taxonomical distribution as uridine kinase, whereas the pyrimidine-nucleoside phosphorylase (2.4.2.2) is only found partially distributed in *Bacteria*, suggesting that this pathway was not present in the LCA. In a third salvage pathway, a single enzymatic step, using uracil phosphoribosyltransferase (2.4.2.9), is required. This enzyme is widely distributed in the three cellular domains, suggesting its presence in the LCA (Figure 6B).

Regarding the salvage routes of ribonucleotides of cytosine, the main pathway occurs in two steps, involving uridine-cytidine kinase (2.7.1.48) and pyrimidine-nucleoside phosphorylase (2.4.2.2), and starting from cytosine as the substrate. Based on their taxonomical distribution, the first enzymatic family, with uridine-cytidine kinase, as previously described does not exhibit a clear distribution pattern to be considered in the LCA, while the second step is only sparsely distributed in *Bacteria*. In addition, there is another cytosine deoxyribonucleotide salvage pathway that starts from deoxycytidine, in which the deoxyadenosine/deoxycytidine kinase (2.7.1.74) participates (Figure 6B), and in turn it is only sparsely distributed in *Eukarya*.

In the salvage pathway of deoxythymine, two enzymatic steps are involved, one with thymidine kinase (2.7.1.21) and the other with thymidine phosphorylase (2.4.2.4), and they start with thymine as the substrate (Figure 6A). The enzyme associated with the first step is partially distributed in *Bacteria* and more sparsely distributed in *Eukarya* and *Archaea*, while the enzyme associated with the second step is sparsely distributed in the three cellular domains. It is difficult to determine whether this pathway was present in the LCA or if its distribution is a reflection of horizontal gene transfer or massive gene loss, due to the poor distribution of the enzymes belonging to this pathway in the three cellular domains.

#### Integrating the evolutionary analysis of the de novo and salvage pathways of pyrimidine metabolism

**Figure 7:**
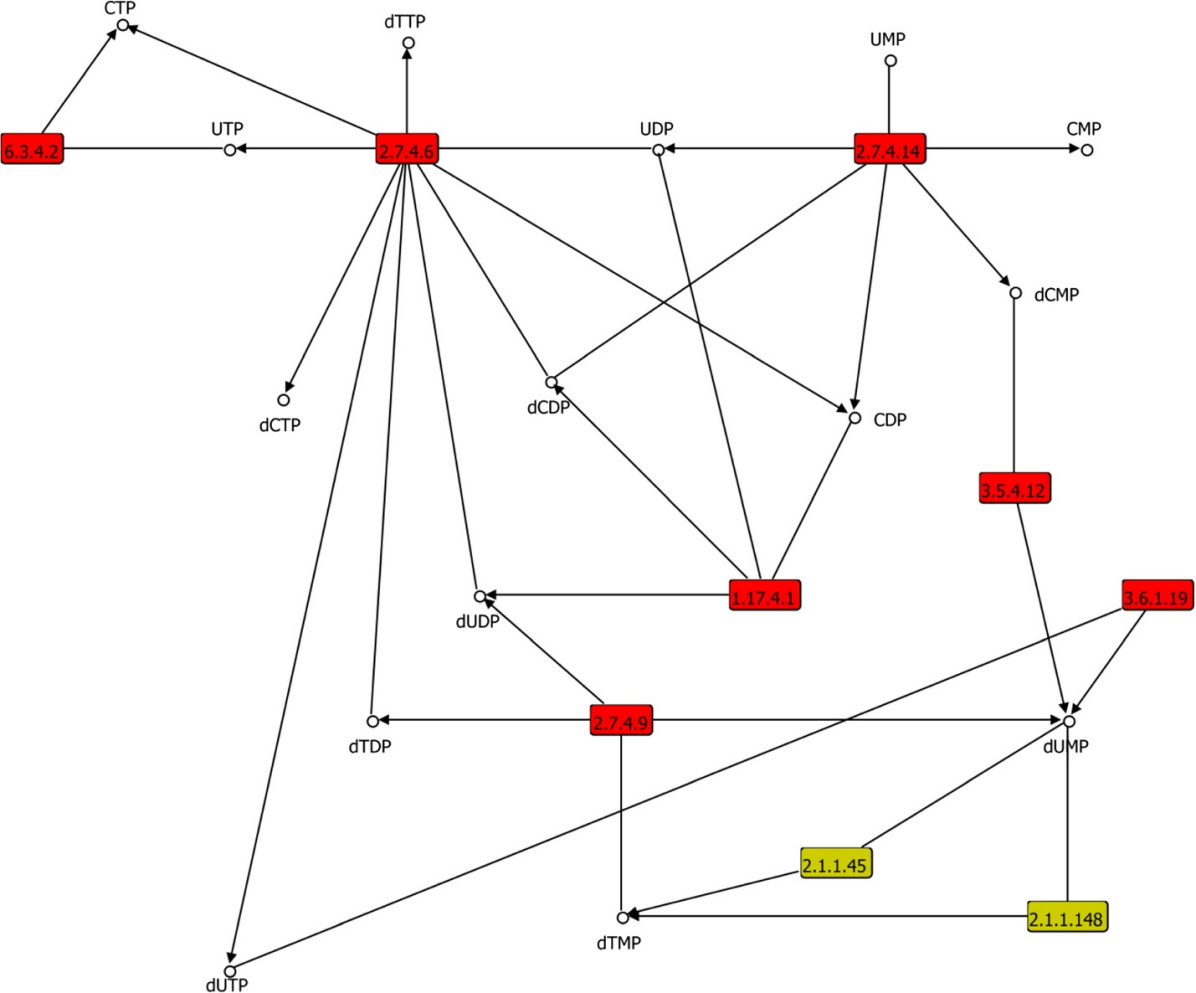
**Possible ancestral route to** ***de novo*** **pyrimidine biosynthesis starting from UMP**. The enzymatic steps associated with the LCA are shown in red. The folate-dependent enzymes for the synthesis of thymine are shown in gold.

Based on the taxonomical distributions of enzymes associated with the *de novo* and salvage pathways for pyrimidine, it was possible to identify, in addition to the *de novo* pathway, a widespread salvage pathway (via 2.4.2.9) for the synthesis of ribonucleotide of uracil (Figure 6B), suggesting that the LCA had two pathways to carry out its synthesis. On the other hand, for the synthesis of uracil deoxyribonucleotides, two main routes, each with one step, have been identified; the first one starts from dCTP, which is converted by the enzyme 3.5.4.13, and the second route starts from dCMP, which is converted by the enzyme linked to 3.5.4.12. The wide taxonomic distribution in the three cellular domains of the 3.5.4.12 enzyme suggests that the second pathway was present in the LCA (Figure 7).

The taxonomical distribution of enzymatic families associated with DHDOS suggests that their divergence correlated with the transition from gram-negative to gram-positive bacteria and was affected by similar evolutionary pressures. The evolutionary pressures could be associated with a variety of environmental changes, such as increased atmospheric oxygen levels, temperature or changes from water to soil habitats. These changes could lead to modifications in the plasma membrane’s chemical properties, resulting in the divergence of family 2 enzymes (1.3.5.2) linked to the cell membrane, which uses quinones from the respiratory chain as electron acceptors, to the family 1 enzymes, which incorporate soluble electron acceptors. This transition from gram-negative to gram-positive was also suggested by Cavalier-Smith [59, 60]; altogether, with our data regarding the taxonomic distribution showing that family 1 is ubiquitous in gram-positive bacteria and archaea, we therefore suggest close evolutionary relationships between archaea and gram-positive bacteria.

Although gram-negative bacteria and eukarya contain the subfamily 2 of DHODS, it is not possible to deduce an evolutionary relationship, as we previously identified between gram-positive bacteria and archaea, because members of this subfamily in eukarya are linked to the mitochondrial membrane. In this regard, the mitochondrial acquisition in *Eukarya* has been described as a probable lateral gene transfer event, as described in the endosymbiont theory [64]. Therefore, our results support the notion of phagocytosis of a gram-negative bacterium by a protoeukaryotic cell, with posterior specialization to mitochondria.

Additionally, *de novo* synthesis of cytosine ribonucleotides and deoxyribonucleotides was probably associated with the LCA. In *de novo* biosynthesis of thymine, the folate-dependent enzymes ThyA and ThyX can catalyze the transformation of this metabolite independently, suggesting that this pathway appeared posterior to the emergence of folate biosynthesis. For enzymes present in the thymine salvage pathway, it was not possible to determine their presence in the LCA.

## Conclusion

The analysis presented here is based on multiple complete genomes belonging to organisms from the three cellular domains, along with current biochemical knowledge. These analyses allowed us to identify issues related to the origin and evolution of nucleotide metabolism. One of our main findings is that we were able to assess the ancestry of some segments of the purine salvage and the *de novo* pathways, which could be complementary and closely related to the LCA of the three cellular domains. Additionally, it was found that a large part of the *de novo* purine branch is widely distributed in *Bacteria*, *Archaea* and *Eukarya*, primarily towards *de novo* biosynthesis of IMP (a key precursor of purines). This branch may have been associated, in the early stages of cell evolution, with the metabolism of thiamine (vitamin B_1_) and posteriorly was complemented by the addition of two new enzymatic steps to complete the IMP biosynthesis pathway by means of the folate-dependent PurH enzyme, giving rise to the modern *de novo* synthesis of purines.

The ancestry and divergence of enzymes associated with these routes provide clues to the environmental changes in the early stages of the emergence of life. Such is the case with the divergence of the enzyme phosphoribosylaminoimidazole carboxylase (4.1.1.21) from N5-carboxyaminoimidazole ribonucleotide mutase (5.4.99.18); the divergence of these two enzymes supports the hypothesis of the origin of life in primitive seas with high levels of HCO_3_^-^. Once the atmosphere was provided with oxygen and the first eukaryotic organisms with mitochondria emerged, the enzyme 5.4.99.18 diverged to 4.1.1.21 through acquisition of a CO_2_ binding site. This divergence process, among other metabolic changes, may have facilitated the emergence of the first eukaryotic multicellular organisms.

In the case of pyrimidines, it was possible for us to infer that the LCA synthesized uracil ribonucleotides, by both the *de novo* and salvage pathways, suggesting that this ribonucleotide could have been involved in a great number of enzymatic functions and/or regulation as a remnant of the RNA world. Additionally, it was possible to associate the synthesis of cytosine and uracil deoxyribonucleotides in the LCA, and once folate biosynthesis was possible, the thymine deoxyribonucleotides emerged due to their enzymatic dependence on the folate precursor. Before the emergence of folate biosynthesis, some variants of these three bases, including cytosine or cytosine-methylated derivatives, based on its similarity to thymine, could have played a role similar to actual thymine in DNA. This inference is supported by the fact that several analogs of cytosine and uracil partially integrate into DNA as replacements for thymine [65–67]. Thus, thymine does not have an active role in the nucleotide transcript and it is more likely to be replaced. This argument is consistent with the evolution of bacterial strains [68], which shows that strains can be generated with the ability to incorporate derivatives of uracil (chloro-uracil) by replacing up to 98% of thymine and maintain cell viability.

In relation to purine synthesis, our results revealed that the biosynthesis of adenine could have been carried out in the LCA by the adenylate kinase. In the case of guanine biosynthesis, we found a complex evolutionary history; for instance, its synthesis has been detected in archaeal organisms, although its gene sequence has not been identified [36]. The fact that we did not find the guanylate kinase bacterial type in *Archaea* suggests three possible scenarios: a) a high sequence divergence between these proteins, b) a masking function for some other enzyme, and/or c) a gene loss or gene nonorthologous displacement. As discussed above, it is likely that the guanylate kinase function has been masked by the adenylate kinase AK6 subfamily type (AK6). This enzyme is homologous to the canonical guanylate kinase of *Eukarya* and *Bacteria* and is probably closer to the possible ancestral enzyme present in the LCA, with promiscuity in the synthesis of NMPs and dNMPs to their respective NDPs and dNDPs. In addition, we found the presence of this multispecific adenylate kinase AK6 subfamily type in *Archaea*, suggesting that this could have been acquired from the LCA with no major posterior changes. In contrast, the guanylate kinase of *Bacteria* and *Eukarya* may have diverged to a greater extent from the adenylate kinase family subsequent to archaeal divergence. The structural similarity of inosine and guanine, together with the remnant affinity of inosine in several proteins such as RNA polymerase, in addition to the taxonomical distribution data that showed a possible LCA route for the synthesis of ITP (Figure 3, magenta circle) by means of a broad-specificity ancestral enzyme similar to the adenylate kinase Ak6 subfamily type, suggest that this base could have played an important role in early cell evolution, as has been previously proposed [41]. The ancestral enzyme might have provided both IDP and GDP for subsequent processing to NTPs, with both playing similar informational process roles in the genetic code based on their structural similarities.

The subsequent specialization of guanylate kinase may have facilitated greater availability of guanine nucleotide to replace inosine, thus avoiding ambiguous rules in DNA replication that would have been achieved with this consolidation stage of the genetic code.

Interestingly, the divergence of the DHOD families in the LCA suggests transitions associated with changes in the cell wall and cell membrane, supporting an order of divergence from cell walls of gram-negative-like organisms and membranes similar to *Eukarya-Bacteria,* towards gram-positive cell wall, and/or membranes similar to Archaea. Since the plasmatic membrane is considered a matter of vertical inheritance, we suggest that the divergence of the family of DHODs can be associated with the divergence of *Archaea* and gram-positive bacteria.

## Methods

### Profiles (RPS-Blast)

In order to select the enzymatic numbers and their corresponding enzymes belonging to nucleotide metabolism, the KEGG [2] and MetaCyc [69] databases were exhaustively explored. In total, 120 enzymatic numbers and their corresponding enzymes were collected. (Additional file 5: Table S3 and Additional file 6: Table S4). In a second step, RPS-Blast profiles were used to search for the occurrence of members of enzyme families in complete genomes. These profiles were extracted from PRIAM, the specialized database for detection of enzymatic sequences [70]. This database encompasses characteristics representative of alignments manually annotated, including members of a particular enzyme family, according to the Enzyme DB [71]. In addition, enzymatic steps can be associated with more than one profile, such as protein complexes, or families of nonenzymatic homologous proteins (analogous enzymes) or steps performed by members of the same families (paralogs). Altogether, profiles were curated manually based on functional annotated domains according to Enzyme DB [71].

### Enzymatic function

For the analysis of enzymatic functions, the best hit for each sequence, with an E-value of ≤10^-10^ and coverage of ≥55% in relation to the profile, was considered. Similar criteria have been previously described for enzymatic annotation of complete genomes [72].

### Taxonomical distributions

In total, 151 sequence profiles associated with 120 enzymatic reactions related to nucleotide metabolism were evaluated in 2,044 complete genomes. The evaluation was based on profile comparisons, using RPS-Blast against the 2,044 complete genomes. Organisms classified as obligate parasites or those organisms with a reduced genome, *i.e.*, less than 1,000 genes, were not considered in this study, with the aim of excluding a possible bias associated with massive gene loss, as previously described [1, 8], leaving us with a total of 1,606 of the 2,044 complete genomes. In order to exclude redundancy in the genomes analyzed, we clustered the organisms based on their taxonomical classifications, in order to obtain a normalized measure of the taxonomical distribution of enzymes according to the following steps. In the first step, we obtained the average presence/absence of enzymes per genus, which in turn was used for the second step, to calculate the average presence/absence of enzymes per Clade. Clades corresponded to the taxonomical categories from the Joint Genome Institute’s Integrated Microbial Genomics. Finally, we consider enzymes as widely distributed as those present in more than 50 percent of the clades of the same cellular domain. Additional file 1: Figure S1 and Additional file 2: Figure S2.

### Identification of structural domains and families

Structural domains of proteins and families were identified using the models deposited in the Superfamily database [73]. In brief, this database contains Hidden Markov models (HMMs) for each superfamily, which are classified according to their structural domains, which in turn are based on the classifications of SCOP [74]; all members of the same superfamily have a common evolutionary origin. Consensus sequences were derived from the alignments (from which profiles were constructed) and used to identify their corresponding superfamilies, using default parameters.

## Author’s contribution

DA-M retrieved all enzymatic repertoires, performed the all sequence analysis and interpreted the results. LS interpreted the results; and EP-R interpreted the results. DA-M and EP-R drafted and wrote the manuscript. All authors read and approved the final manuscript.

## Electronic supplementary material

Additional file 1: Figure S1: Average taxonomic distribution of purine metabolism enzymes distributed across the three domains of life. The taxonomic distribution for enzymes catalyzing the purine metabolism (vertical labels) was computed by searching for their ortholog distribution across diverse taxonomic groups (*Archaea, Bacteria and Eukarya)* (horizontal labels). Enzymes are sorted in terms of their E.C. number. Some identical E.C. numbers and superfamily assignation are complementary according to PRIAM db. The “S” notation defines protein subunits; “AN” defines ANalogous enzymes, those defined as enzymes with the same E.C. number and the different superfamily classification, suggesting different evolutionary origin. The colors are in rainbow scale, where dark blue are those enzymes with low average (0) whereas red denotes high average values (1). (TIFF 311 KB)

Additional file 2: Figure S2: Average taxonomic distribution of pyrimidine metabolism enzymes. Labels and colors are as in Additional file 1: Figure S1. In asterisks are indicated those enzymes common to purine and pyrimidine metabolisms. (TIFF 433 KB)

Additional file 3: Table S1: Matrix used to construct the average taxonomic distribution. Purine metabolism. Column 1 shows the E.C. numbers; columns 2 to 76 correspond to the taxonomical divisions analyzed here. Each cell includes normalized values as described in material and methods. (XLSX 35 kb). (XLSX 35 KB)

Additional file 4: Table S2: Matrix used to construct the average taxonomic distribution. Pyrimidine metabolism. Column 1 shows the E.C. numbers; columns 2 to 76 correspond to the taxonomical divisions analyzed here. Each cell includes normalized values as described in material and methods. (XLSX 34 KB)

Additional file 5: Table S3: Enzymes analyzed. Purine metabolism (Map 00230). Column 1 denotes the E.C. Number; column 2, the accepted name; column 3, reactions associated to each E. C. number in the purine metabolism; and column 4, the identification code on KEGG database. (DOCX 32 KB)

Additional file 6: Table S4: Enzymes analyzed. Pyrimidine metabolism (Map 00240). Column 1 denotes the E.C. Number; column 2, the accepted name; column 3, reactions associated to each E. C. number in the pyrimidine metabolism; and column 4, the identification code on KEGG database. (DOCX 35 KB)
