# Supplementary figures and images for: Comparative genomics of nucleotide metabolism: a tour to the past of the three cellular domains of life
Source: BMC Genomics. 2014 Sep 17;15(1):800. doi: 10.1186/1471-2164-15-800 (PMC4177761; doi:10.1186/1471-2164-15-800)

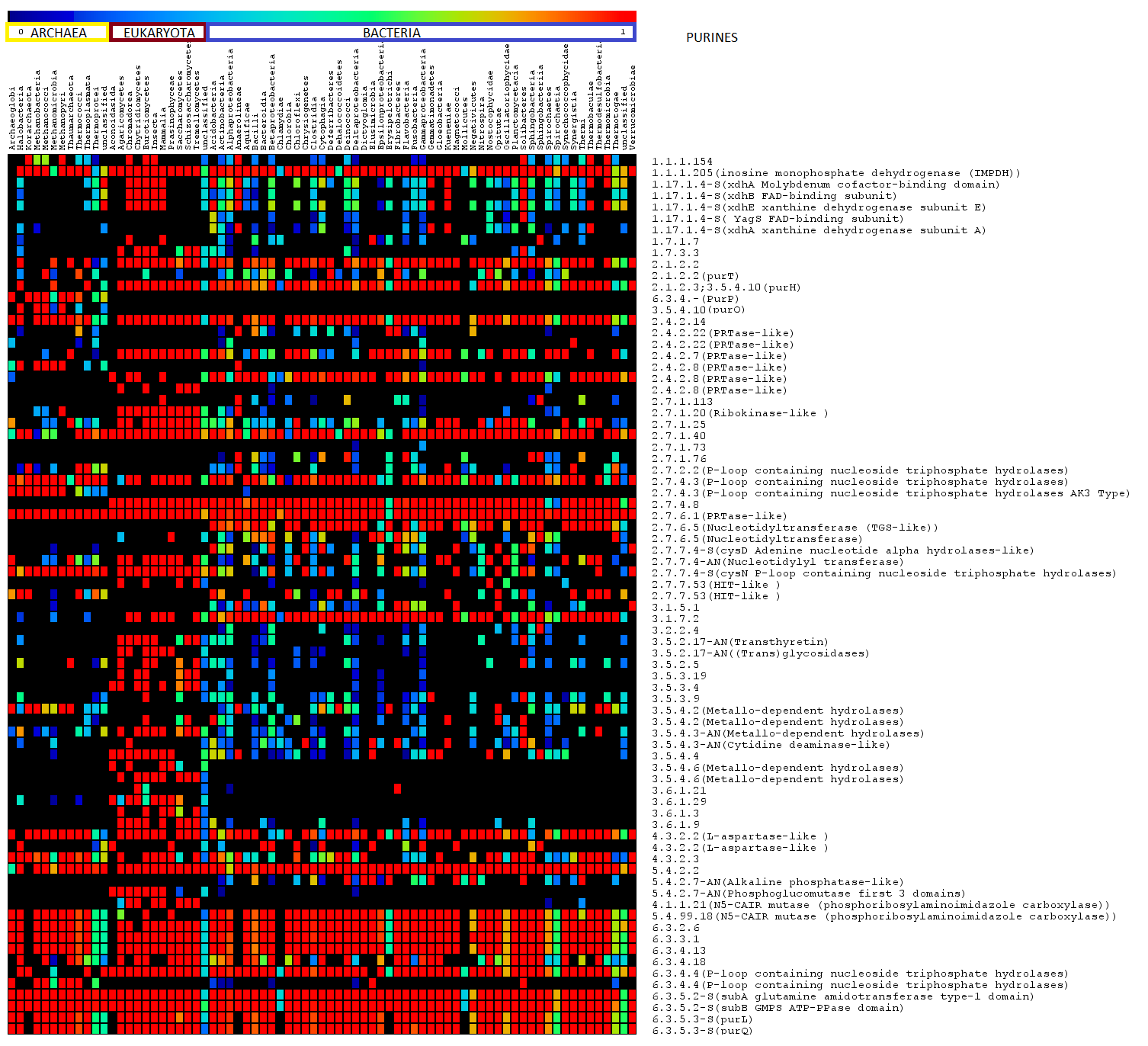

Supplement: Supplementary file 1 — Additional file 1: Figure S1: Average taxonomic distribution of purine metabolism enzymes distributed across the three domains of life. The taxonomic distribution for enzymes catalyzing the purine metabolism (vertical labels) was computed by searching for their ortholog distribution across diverse taxonomic groups (Archaea, Bacteria and Eukarya) (horizontal labels). Enzymes are sorted in terms of their E.C. number. Some identical E.C. numbers and superfamily assignation are complementary according to PRIAM db. The “S” notation defines protein subunits; “AN” defines ANalogous enzymes, those defined as enzymes with the same E.C. number and the different superfamily classification, suggesting different evolutionary origin. The colors are in rainbow scale, where dark blue are those enzymes with low average (0) whereas red denotes high average values (1). (TIFF 311 KB) [file 12864_2014_6481_MOESM1_ESM.tiff]

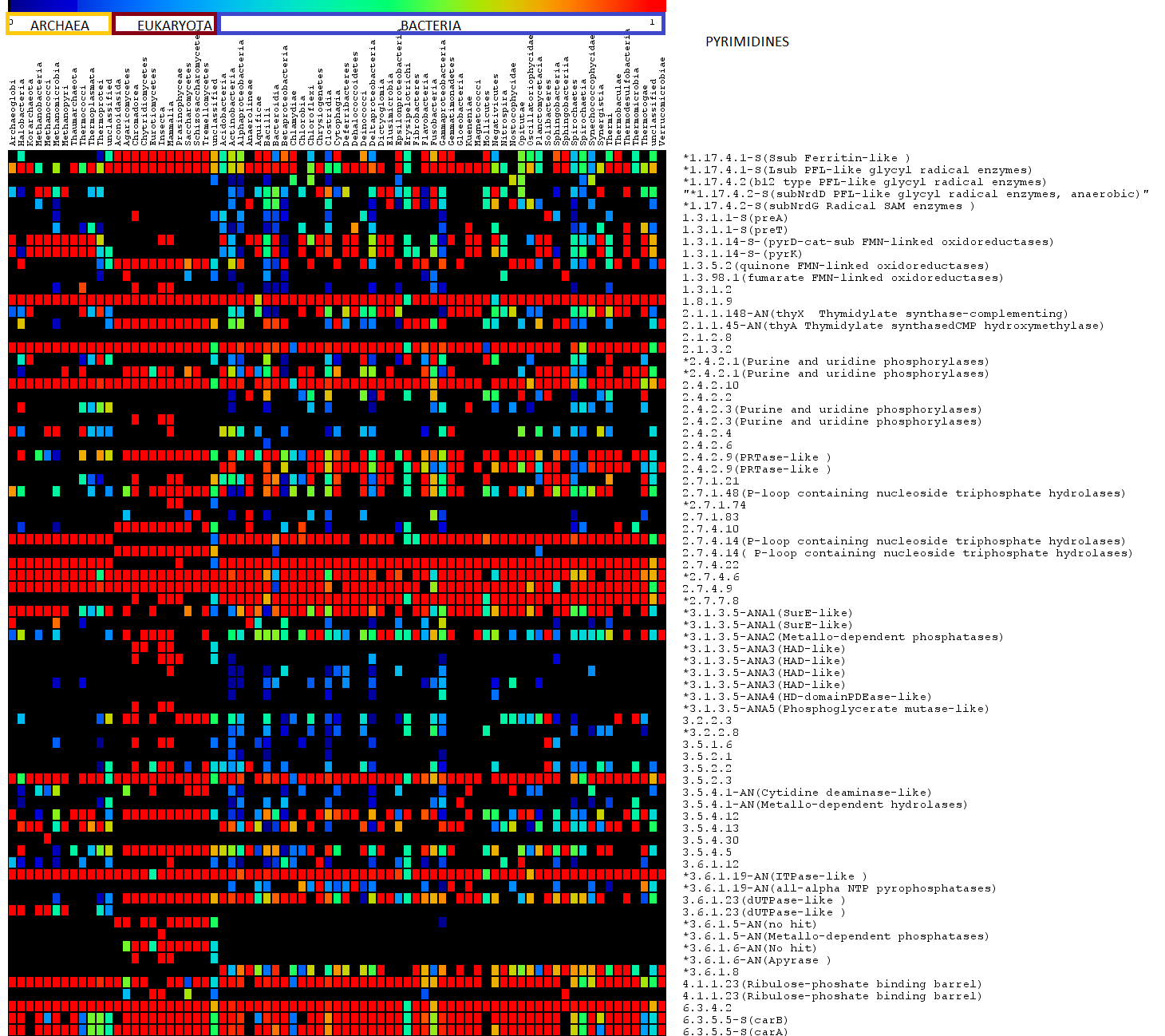

Supplement: Supplementary file 2 — Additional file 2: Figure S2: Average taxonomic distribution of pyrimidine metabolism enzymes. Labels and colors are as in Additional file 1: Figure S1. In asterisks are indicated those enzymes common to purine and pyrimidine metabolisms. (TIFF 433 KB) [file 12864_2014_6481_MOESM2_ESM.tiff]
