# Supplementary material for: Comparative genomics of nucleotide metabolism: a tour to the past of the three cellular domains of life
Source: BMC Genomics. 2014 Sep 17;15(1):800. doi: 10.1186/1471-2164-15-800 (PMC4177761; doi:10.1186/1471-2164-15-800)
Supplement: Supplementary file 5 — Additional file 5: Table S3: Enzymes analyzed. Purine metabolism (Map 00230). Column 1 denotes the E.C. Number; column 2, the accepted name; column 3, reactions associated to each E. C. number in the purine metabolism; and column 4, the identification code on KEGG database. (DOCX 32 KB) [file 12864_2014_6481_MOESM5_ESM.docx]

**Supplementary material associated to the manuscript “*Comparative genomics of nucleotide metabolism: A tour to the past of the three cellular domains of life”* by D*. Armenta-Medina, L. Segovia and E. Perez-Rueda***

**Table S1. Enzymes analyzed. Purine metabolism (Map 00230).**

| **E. C. Number** | **Accepted name** | **Reactions** | **Reaction_Code_KEGG** |
| --- | --- | --- | --- |
| EC:1.1.1.154 | Ureidoglycolate dehydrogenase | (-)-Ureidoglycolate + NAD+ <=> Oxalureate + NADH + H+  (-)-Ureidoglycolate + NADP+ <=> Oxalureate + NADPH + H+ | rn:R02935  rn:R02936 |
| EC:1.1.1.205 | IMP dehydrogenase | IMP + NAD+ + H2O <=> Xanthosine 5'-phosphate + NADH + H+ | rn:R01130 |
| EC:1.17.1.4 | Xanthine dehydrogenase molybdenum-binding subunit | Hypoxanthine + NAD+ + H2O <=> Xanthine + NADH + H+  Hypoxanthine + Oxygen + H2O <=> Xanthine + Hydrogen peroxide  Xanthine + NAD+ + H2O <=> Urate + NADH + H+  Xanthine + H2O + Oxygen <=> Urate + Hydrogen peroxide | rn:R01768  rn:R01769  rn:R02103  rn:R02107 |
| EC:1.17.3.2 | Xanthine dehydrogenase/oxidaseX | Hypoxanthine + NAD+ + H2O <=> Xanthine + NADH + H+  Hypoxanthine + Oxygen + H2O <=> Xanthine + Hydrogen peroxide  Xanthine + NAD+ + H2O <=> Urate + NADH + H+  Xanthine + H2O + Oxygen <=> Urate + Hydrogen peroxide | rn:R01768  rn:R01769  rn:R02103  rn:R02107 |
| EC:1.7.1.7 | GMP reductase | IMP + NH3 + NADP+ <=> GMP + NADPH + H+ | rn:R01134 |
| EC:1.7.3.3 | Urate oxidase | Urate + Oxygen + H2O <=> 5-Hydroxyisourate + Hydrogen peroxide | rn:R02106 |
| EC:2.1.2.2 | Phosphoribosylglycinamide formyltransferase 1 (PurN) and phosphoribosylglycinamide formyltransferase 2 (PurT) | 10-Formyltetrahydrofolate + 5'-Phosphoribosylglycinamide <=> Tetrahydrofolate + 5'-Phosphoribosyl-N-formylglycinamide (purN)  Formate + ATP + 5'-phospho-ribosylglycinamide = 5'-phosphoribosyl-N-formylglycinamide + ADP + diphosphate (purT) | rn:not assigned  rn:R04325 |
| EC:2.1.2.3(PURH) | Phosphoribosylaminoimidazolecarboxamide formyltransferase | IMP + H2O <=> 1-(5'-Phosphoribosyl)-5-formamido-4-imidazolecarboxamide | rn:R01127 |
|  | IMP cyclohydrolase | 10-Formyltetrahydrofolate + 1-(5'-Phosphoribosyl)-5-amino-4-imidazolecarboxamide <=> Tetrahydrofolate + 1-(5'-Phosphoribosyl)-5-formamido-4-imidazolecarboxamide | rn:R04560 |
| EC:2.4.2.14 | Amidophosphoribosyltransferase | 5-Phosphoribosylamine + Diphosphate + L-Glutamate <=> L-Glutamine + 5-Phospho-alpha-D-ribose 1-diphosphate + H2O | rn:R01072 |
| EC:2.4.2.22 | Purine-nucleoside phosphorylase | GMP + Diphosphate <=> Guanine + 5-Phospho-alpha-D-ribose 1-diphosphate  Xanthosine 5'-phosphate + Diphosphate <=> Xanthine + 5-Phospho-alpha-D-ribose 1-diphosphate | rn:R01229  rn:R02142 |
| EC:2.4.2.7 | Adenine phosphoribosyltransferase | AMP + Diphosphate <=> Adenine + 5-Phospho-alpha-D-ribose 1-diphosphate  GMP + Diphosphate <=> Guanine + 5-Phospho-alpha-D-ribose 1-diphosphate  1-(5'-Phosphoribosyl)-5-amino-4-imidazolecarboxamide + Diphosphate <=> 5-Amino-4-imidazolecarboxyamide + 5-Phospho-alpha-D-ribose 1-diphosphate | rn:R00190  rn:R01229  rn:R04378 |
| EC:2.4.2.8 | Hypoxanthine phosphoribosyltransferase | AMP + Diphosphate <=> Adenine + 5-Phospho-alpha-D-ribose 1-diphosphate  IMP + Diphosphate <=> Hypoxanthine + 5-Phospho-alpha-D-ribose 1-diphosphate  GMP + Diphosphate <=> Guanine + 5-Phospho-alpha-D-ribose 1-diphosphate  Xanthosine 5'-phosphate + Diphosphate <=> Xanthine + 5-Phospho-alpha-D-ribose 1-diphosphate | rn:R00190  rn:R01132  rn:R01229  rn:R02142 |
| EC:2.7.1.113 | Deoxyguanosine kinase | ATP + Deoxyguanosine <=> ADP + dGMP | rn:R01967 |
| EC:2.7.1.20 | Adenosine kinase | ATP + Adenosine <=> ADP + AMP | rn:R00185 |
| EC:2.7.1.25 | Adenylylsulfate kinase | ATP + Adenylyl sulfate <=> ADP + 3'-Phosphoadenylyl sulfate  ATP + Sulfate <=> Diphosphate + Adenylyl sulfate | rn:R00509  rn:R00529 |
| EC:2.7.1.40 | Pyruvate kinase | ATP + Pyruvate <=> ADP + Phosphoenolpyruvate  GTP + Pyruvate <=> GDP + Phosphoenolpyruvate  CTP + Pyruvate <=> CDP + Phosphoenolpyruvate  UTP + Pyruvate <=> UDP + Phosphoenolpyruvate  ITP + Pyruvate <=> IDP + Phosphoenolpyruvate  dATP + Pyruvate <=> dADP + Phosphoenolpyruvate  dGTP + Pyruvate <=> dGDP + Phosphoenolpyruvate  Nucleoside triphosphate + Pyruvate <=> NDP + Phosphoenolpyruvate | rn:R00200  rn:R00430  rn:R00572  rn:R00659  rn:R00724  rn:R01138  rn:R01858  rn:R02320 |
| EC:2.7.1.73 | Inosine kinase | ATP + Inosine <=> ADP + IMP  ATP + Guanosine <=> ADP + GMP | rn:R01131  rn:R01228 |
| EC:2.7.1.76 | Deoxyadenosine kinase | ATP + Deoxyadenosine <=> ADP + dAMP | rn:R02089 |
| EC:2.7.2.2 | Darbamate kinase | ATP + NH3 + CO2 <=> ADP + Carbamoyl phosphate | rn:R00150 |
| EC:2.7.4.3 | Adenylate kinase | ATP + AMP <=> 2 ADP  ATP + dAMP <=> ADP + dADP | rn:R00127  rn:R01547 |
| EC:2.7.4.8 | Guanylate kinase | ATP + GMP <=> ADP + GDP  ATP + dGMP <=> ADP + dGDP | rn:R00332  rn:R02090 |
| EC:2.7.6.1 | Ribose-phosphate pyrophosphokinase | ATP + D-Ribose 5-phosphate <=> AMP + 5-Phospho-alpha-D-ribose 1-diphosphate | rn:R01049 |
| EC:2.7.6.5 | GTP pyrophosphokinase | ATP + GTP <=> AMP + Guanosine 3'-diphosphate 5'-triphosphate | rn:R00429 |
| EC:2.7.7.4 | Bifunctional enzyme CysN/CysC | ATP + Sulfate <=> Diphosphate + Adenylyl sulfate | rn:R00529 |
| EC:2.7.7.53 | ATP adenylyltransferase | ADP + ATP <=> Orthophosphate + P1,P4-Bis(5'-adenosyl) tetraphosphate  Adenylyl sulfate + ATP <=> Sulfate + P1,P4-Bis(5'-adenosyl) tetraphosphate | rn:R00126  rn:R01618 |
| EC:3.1.5.1 | dGTPase | dGTP + H2O <=> Deoxyguanosine + Triphosphate | rn:R01856 |
| EC:3.1.7.2 | Guanosine-3',5'-bis(diphosphate) 3'-pyrophosphohydrolase | Guanosine 3',5'-bis(diphosphate) + H2O <=> GDP + Diphosphate | rn:R00336 |
| EC:3.2.2.4 | AMP nucleosidase | AMP + H2O <=> Adenine + D-Ribose 5-phosphate | rn:R00182 |
| EC:3.5.3.19 Transferred to 3.5.1.116 | Ureidoglycolate amidohydrolase | (-)-Ureidoglycolate + H2O <=> Glyoxylate + 2 NH3 + CO2 | rn:R00469 |
| EC:3.5.2.17 | 5-hydroxyisourate hydrolase | 5-Hydroxyisourate + H2O <=> 5-Hydroxy-2-oxo-4-ureido-2,5-dihydro-1H-imidazole-5-carboxylate | rn:R06601 |
| EC:3.5.2.5 | Allantoinase | (S)(+)-Allantoin + H2O <=> Allantoate | rn:R02425 |
| EC:3.5.3.4 | Allantoicase | Allantoate + H2O <=> (-)-Ureidoglycolate + Urea | rn:R02422 |
| EC:3.5.3.9 | Allantoate deiminase | Allantoate + H2O <=> (S)-Ureidoglycine + NH3 + CO2 | rn:R02423 |
| EC:3.5.4.10 | IMP cyclohydrolase | IMP + H2O <=> 1-(5'-Phosphoribosyl)-5-formamido-4-imidazolecarboxamide | rn:R01127 |
| EC:3.5.4.2 | Adenine deaminase | Adenine + H2O <=> Hypoxanthine + NH3 | rn:R01244 |
| EC:3.5.4.3 | Guanine deaminase | Guanine + H2O <=> Xanthine + NH3 | rn:R01676 |
| EC:3.5.4.4 | Adenosine deaminase | Adenosine + H2O <=> Inosine + NH3  Deoxyadenosine + H2O <=> Deoxyinosine + NH3 | rn:R01560  rn:R02556 |
| EC:3.5.4.6 | AMP deaminase | AMP + H2O <=> IMP + NH3 | rn:R00181 |
| EC:3.6.1.21 | ADP-sugar diphosphatase | ADP-ribose + H2O <=> alpha-D-Ribose 1-phosphate + AMP | rn:R01885 |
| EC:3.6.1.29 | bis(5'-adenosyl)-triphosphatase | P1,P3-Bis(5'-adenosyl) triphosphate + H2O <=> ADP + AMP | rn:R00187 |
| EC:3.6.1.3 | Adenosinetriphosphatase | ATP + H2O <=> ADP + Orthophosphate | rn:R00086 |
| EC:3.6.1.6 | Nucleoside diphosphate phosphatase | UDP + H2O <=> UMP + Orthophosphate  GDP + H2O <=> GMP + Orthophosphate  NDP + H2O <=> Nucleotide + Orthophosphate  IDP + H2O <=> IMP + Orthophosphate | rn:R00155  rn:R00328  rn:R00329  rn:R00961 |
| EC:3.6.1.9 | Nucleotide diphosphatase | Dinucleotide + H2O <=> 2 Mononucleotide | rn:R00056 |
| EC:4.3.2.2 | Adenylosuccinate lyase | N6-(1,2-Dicarboxyethyl)-AMP <=> Fumarate + AMP  1-(5'-Phosphoribosyl)-5-amino-4-(N-succinocarboxamide)-imidazole <=> Fumarate + 1-(5'-Phosphoribosyl)-5-amino-4-imidazolecarboxamide | rn:R01083  rn:R04559 |
| EC:4.3.2.3 | Ureidoglycolate lyase | (-)-Ureidoglycolate <=> Glyoxylate + Urea | rn:R00776 |
| EC:5.4.2.2 | Phosphoglucomutase | alpha-D-Ribose 1-phosphate <=> D-Ribose 5-phosphate | rn:R01057 |
| EC:5.4.2.7 | Phosphopentomutase | alpha-D-Ribose 1-phosphate <=> D-Ribose 5-phosphate | rn:R01057 |
| EC:4.1.1.21 | Phosphoribosylaminoimidazole carboxylase | 1-(5-Phospho-D-ribosyl)-5-amino-4-imidazolecarboxylate <=> Aminoimidazole ribotide + CO2 | rn:R04209 |
| EC:5.4.99.18 | 5-(carboxyamino)imidazole ribonucleotide mutase | 5-Carboxyamino-1-(5-phospho-D-ribosyl)imidazole <=> 1-(5-Phospho-D-ribosyl)-5-amino-4-imidazolecarboxylate | rn:R07405 |
| EC:6.3.2.6 | Phosphoribosylaminoimidazolesuccinocarboxamide synthase | ATP + 1-(5-Phospho-D-ribosyl)-5-amino-4-imidazolecarboxylate + L-Aspartate <=> ADP + Orthophosphate + 1-(5'-Phosphoribosyl)-5-amino-4-(N-succinocarboxamide)-imidazole | rn:R04591 |
| EC:6.3.3.1 | Phosphoribosylaminoimidazole synthetase | ATP + 2-(formamido)-N1-(5-phospho-D-ribosyl)acetamidine = ADP + phosphate + 5-amino-1-(5-phospho-D-ribosyl)imidazole | rn:R04208 |
| EC:6.3.4.13 | Phosphoribosylglycinamide synthetase | ATP + 5-phospho-D-ribosylamine + glycine = ADP + phosphate + N1-(5-phospho-D-ribosyl)glycinamide | rn:R04144 |
| EC:6.3.4.18 | 5-(carboxyamino)imidazole ribonucleotide synthase | ATP + 5-amino-1-(5-phospho-D-ribosyl)imidazole + HCO3- = ADP + phosphate + 5-carboxyamino-1-(5-phospho-D-ribosyl)imidazole | rn:R07404 |
| EC:6.3.4.23 | Formate---phosphoribosylaminoimidazolecarboxamide ligase. PurP. | ATP + formate + 5-amino-1-(5-phospho-D-ribosyl)imidazole-4-carboxamide = ADP + phosphate + 5-formamido-1-(5-phospho-D-ribosyl)imidazole-4-carboxamide | rn:R06975 |
| EC:6.3.4.4 | Adenylosuccinate synthase | GTP + IMP + L-aspartate = GDP + phosphate + N6-(1,2-dicarboxyethyl)-AMP | rn:R01135 |
| EC:6.3.5.2 | GMP synthase (glutamine-hydrolysing) | ATP + Xanthosine 5'-phosphate + Ammonia <=> AMP + Diphosphate + GMP  ATP + Xanthosine 5'-phosphate + L-Glutamine + H2O <=> AMP + Diphosphate + GMP + L-Glutamate | rn:R01230  rn:R01231 |
| EC:6.3.5.3 | Phosphoribosylformylglycinamidine synthase | ATP + N2-formyl-N1-(5-phospho-D-ribosyl)glycinamide + L-glutamine + H2O = ADP + phosphate + 2-(formamido)-N1-(5-phospho-D-ribosyl)acetamidine + L-glutamate | rn:R04463 |
