# Supplementary material for: Comparative genomics of nucleotide metabolism: a tour to the past of the three cellular domains of life
Source: BMC Genomics. 2014 Sep 17;15(1):800. doi: 10.1186/1471-2164-15-800 (PMC4177761; doi:10.1186/1471-2164-15-800)
Supplement: Supplementary file 6 — Additional file 6: Table S4: Enzymes analyzed. Pyrimidine metabolism (Map 00240). Column 1 denotes the E.C. Number; column 2, the accepted name; column 3, reactions associated to each E. C. number in the pyrimidine metabolism; and column 4, the identification code on KEGG database. (DOCX 35 KB) [file 12864_2014_6481_MOESM6_ESM.docx]

**Supplementary material associated to the manuscript “*Comparative genomics of nucleotide metabolism: A tour to the past of the three cellular domains of life”* by D*. Armenta-Medina, L. Segovia and E. Perez-Rueda***

**Table S2. Enzymes analyzed. Pyrimidine metabolism (Map 00240).**

| **E. C. Number** | **Accepted name** | **Reactions** | **Reaction_Code_KEGG** |
| --- | --- | --- | --- |
| EC:1.17.4.1 | Ribonucleotide reductase, class II | 2'-Deoxyribonucleoside diphosphate + Thioredoxin disulfide + H2O <=> Ribonucleoside diphosphate + Thioredoxin  dADP + Thioredoxin disulfide + H2O <=> Thioredoxin + ADP  dCDP + Thioredoxin disulfide + H2O <=> Thioredoxin + CDP  dGDP + Thioredoxin disulfide + H2O <=> GDP + Thioredoxin  dUDP + Thioredoxin disulfide + H2O <=> Thioredoxin + UDP | rn:R04294  rn:R02017  rn:R02024  rn:R02019  rn:R02018 |
| EC:1.17.4.2 | Ribonucleoside-triphosphate reductase | 2'-Deoxyribonucleoside triphosphate + Thioredoxin disulfide + H2O <=> Ribonucleoside triphosphate + Thioredoxin  dATP + Thioredoxin disulfide + H2O <=> ATP + Thioredoxin  dGTP + Thioredoxin disulfide + H2O <=> GTP + Thioredoxin  dCTP + Thioredoxin disulfide + H2O <=> CTP + Thioredoxin  dUTP + Thioredoxin disulfide + H2O <=> UTP + Thioredoxin | rn:R04315  rn:R02014  rn:R02020  rn:R02022  rn:R02023 |
| EC:1.3.1.1 | Dihydropyrimidine dehydrogenase (NAD+) | 5,6-Dihydrothymine + NAD+ <=> Thymine + NADH + H+  5,6-Dihydrouracil + NAD+ <=> Uracil + NADH + H+ | rn:R01414  rn:R00977 |
| EC:1.3.1.14 | Dihydroorotate dehydrogenase (NAD+) | (S)-Dihydroorotate + NAD+ <=> Orotate + H+ + NADH | rn:R01869 |
| EC:1.3.5.2 | Dihydroorotate dehydrogenase | (S)-Dihydroorotate + Quinone <=> Orotate + Hydroquinone | rn:R01868 |
| EC:1.3.98.1 | Dihydroorotate dehydrogenase (fumarate) | (S)-Dihydroorotate + Oxygen <=> Orotate + Hydrogen peroxide | rn:R01867 |
| EC:1.3.1.2 | Dihydropyrimidine dehydrogenase (NADP+) | 5,6-Dihydrouracil + NADP+ <=> Uracil + NADPH + H+  5,6-Dihydrothymine + NADP+ <=> Thymine + NADPH + H+ | rn:R00978  rn:R01415 |
| EC:1.8.1.9 | Thioredoxin reductase (NADPH) | Thioredoxin + NADP+ <=> Thioredoxin disulfide + NADPH + H+ | rn:R02016 |
| EC:2.1.1.148 | Thymidylate synthase (FAD) | 5,10-Methylenetetrahydrofolate + dUMP + FADH2 <=> Tetrahydrofolate + dTMP + FAD | rn:R06613 |
| EC:2.1.1.45 | Thymidylate synthase | dUMP + 5,10-Methylenetetrahydrofolate <=> Dihydrofolate + dTMP | rn:R02101 |
| EC:2.1.2.8 | Deoxycytidylate 5-hydroxymethyltransferase | 5,10-Methylenetetrahydrofolate + H2O + dCMP <=> Tetrahydrofolate + 5-Hydroxymethyldeoxycytidylate | rn:R01669 |
| EC:2.1.3.2 | Aspartate carbamoyltransferase | Carbamoyl phosphate + L-Aspartate <=> Orthophosphate + N-Carbamoyl-L-aspartate | rn:R01397 |
| EC:2.4.2.1 | Purine-nucleoside phosphorylase | Purine deoxyribonucleoside + Orthophosphate <=> Purine + 2-Deoxy-D-ribose 1-phosphate | rn:R10244 |
| EC:2.4.2.10 | Orotate phosphoribosyltransferase | Orotidine 5'-phosphate + Diphosphate <=> Orotate + 5-Phospho-alpha-D-ribose 1-diphosphate | rn:R01870 |
| EC:2.4.2.2 | Pyrimidine-nucleoside phosphorylase | Cytidine + Orthophosphate <=> Cytosine + alpha-D-Ribose 1-phosphate  Deoxyuridine + Orthophosphate <=> Uracil + 2-Deoxy-D-ribose 1-phosphate  Thymidine + Orthophosphate <=> Thymine + 2-Deoxy-D-ribose 1-phosphate  Uridine + Orthophosphate <=> Uracil + alpha-D-Ribose 1-phosphate | rn:R02296  rn:R02484  rn:R01570  rn:R01876 |
| EC:2.4.2.3 | Uridine phosphorylase | Deoxyuridine + Orthophosphate <=> Uracil + 2-Deoxy-D-ribose 1-phosphate  Uridine + Orthophosphate <=> Uracil + alpha-D-Ribose 1-phosphate | rn:R02484  rn:R01876 |
| EC:2.4.2.4 | Thymidine phosphorylase | Deoxyguanosine + Orthophosphate <=> Guanine + 2-Deoxy-D-ribose 1-phosphate  Deoxyinosine + Orthophosphate <=> Hypoxanthine + 2-Deoxy-D-ribose 1-phosphate  Deoxyuridine + Orthophosphate <=> Uracil + 2-Deoxy-D-ribose 1-phosphate  Thymidine + Orthophosphate <=> Thymine + 2-Deoxy-D-ribose 1-phosphate | rn:R01969  rn:R02748  rn:R02484  rn:R01570 |
| EC:2.4.2.6 | Nucleoside deoxyribosyltransferase | Deoxynucleoside + Base <=> Deoxynucleoside + Base  Thymidine + Base <=> Deoxynucleoside + Thymine | rn:R04168  rn:R02806 |
| EC:2.4.2.9 | Uracil phosphoribosyltransferase | UMP + Diphosphate <=> Uracil + 5-Phospho-alpha-D-ribose 1-diphosphate | rn:R00966 |
| EC:2.7.1.21 | Thymidine kinase | ATP + Deoxyuridine <=> ADP + dUMP  ATP + Thymidine <=> ADP + dTMP | rn:R02099  rn:R01567 |
| EC:2.7.1.48 | Uridine kinase | UTP + Cytidine <=> UDP + CMP  UTP + Uridine <=> UDP + UMP | rn:R00516  rn:R00967 |
| EC:2.7.1.74 | Deoxycitidine kinase | ATP + Adenosine <=> ADP + AMP  ATP + Deoxyadenosine <=> ADP + dAMP  ATP + Deoxycytidine <=> ADP + dCMP  Nucleoside triphosphate + Deoxycytidine <=> NDP + dCMP | rn:R00185  rn:R02089  rn:R01666  rn:R02321 |
| EC:2.7.1.83 | Pseudouridine kinase | ATP + Pseudouridine <=> ADP + Pseudouridine 5'-phosphate  Uracil + D-Ribose 5-phosphate <=> Pseudouridine 5'-phosphate + H2O | rn:R03315  rn:R01055 |
| EC:2.7.4.10 | Nucleoside-triphosphate--adenylate kinase | Nucleoside triphosphate + AMP <=> NDP + ADP  UTP + AMP <=> UDP + ADP | rn:R00333  rn:R00157 |
| EC:2.7.4.14 | Cytidylate kinase | Diphosphate + Pantothenate  ATP + CMP <=> ADP + CDP  ATP + UMP <=> ADP + UDP  ATP + dCMP <=> ADP + dCDP | rn:R00512  rn:R00158  rn:R01665 |
| EC:2.7.4.22 | UMP kinase | ATP + UMP <=> ADP + UDP | rn:R00158 |
| EC:2.7.4.6 | Nucleoside-diphosphate kinase | ATP + ADP <=> ADP + ATP  ATP + CDP <=> ADP + CTP  ATP + GDP <=> ADP + GTP  ATP + IDP <=> ADP + ITP  ATP + NDP <=> ADP + Nucleoside triphosphate  ATP + UDP <=> ADP + UTP  ATP + dADP <=> ADP + dATP  ATP + dCDP <=> ADP + dCTP  ATP + dGDP <=> ADP + dGTP  ATP + dIDP <=> ADP + dITP  ATP + dTDP <=> ADP + dTTP  ATP + dUDP <=> ADP + dUTP | rn:R00124  rn:R00570  rn:R00330  rn:R00722  rn:R00331  rn:R00156  rn:R01137  rn:R02326  rn:R01857  rn:R03530  rn:R02093  rn:R02331 |
| EC:2.7.4.9 | dTMP kinase | ATP + dTMP <=> ADP + dTDP  ATP + dUMP <=> ADP + dUDP | rn:R02094  rn:R02098 |
| EC:2.7.7.8 | Polyribonucleotide nucleotidyltransferase | RNA + Orthophosphate <=> RNA + ADP  RNA + Orthophosphate <=> RNA + CDP  RNA + Orthophosphate <=> RNA + GDP  RNA + Orthophosphate <=> RNA + NDP  RNA + Orthophosphate <=> RNA + UDP | rn:R00437  rn:R00440  rn:R00439  rn:R07282  rn:R00438 |
| EC:3.1.3.5 | 5'-nucleotidase | 5'-Ribonucleotide + H2O <=> Ribonucleoside + Orthophosphate  AMP + H2O <=> Adenosine + Orthophosphate  CMP + H2O <=> Cytidine + Orthophosphate  GMP + H2O <=> Guanosine + Orthophosphate  IMP + H2O <=> Inosine + Orthophosphate  UMP + H2O <=> Uridine + Orthophosphate  Xanthosine 5'-phosphate + H2O <=> Xanthosine + Orthophosphate  dAMP + H2O <=> Deoxyadenosine + Orthophosphate  dCMP + H2O <=> Deoxycytidine + Orthophosphate  dGMP + H2O <=> Deoxyguanosine + Orthophosphate  dTMP + H2O <=> Thymidine + Orthophosphate  dUMP + H2O <=> Deoxyuridine + Orthophosphate | rn:R07297  rn:R00183  rn:R00511  rn:R01227  rn:R01126  rn:R00963  rn:R02719  rn:R02088  rn:R01664  rn:R01968  rn:R01569  rn:R02102 |
| EC:3.2.2.3 | Uridine nucleosidase | Uridine + H2O <=> Uracil + D-Ribose | rn:R01080 |
| EC:3.2.2.8 | Ribosylpyrimidine nucleosidase | Adenosine + H2O <=> Adenine + D-Ribose  Cytidine + H2O <=> Cytosine + D-Ribose  Guanosine + H2O <=> Guanine + D-Ribose  Inosine + H2O <=> Hypoxanthine + D-Ribose  Pyrimidine nucleoside + H2O <=> Pyrimidine + D-Ribose + H+ | rn:R01245  rn:R02137  rn:R01677  rn:R01770  rn:R02172 |
| EC:3.5.1.6 | Beta-ureidopropionase | 3-Ureidoisobutyrate + H2O <=> 3-Aminoisobutyric acid + CO2 + NH3  3-Ureidopropionate + H2O <=> beta-Alanine + CO2 + NH3 | rn:R04666  rn:R00905 |
| EC:3.5.2.1 | Barbiturase | Barbiturate + H2O <=> 3-Oxo-3-ureidopropanoate | rn:R02139 |
| EC:3.5.2.2 | Dihydropyrimidinase | 5,6-Dihydrothymine + H2O <=> 3-Ureidoisobutyrate  5,6-Dihydrouracil + H2O <=> 3-Ureidopropionate | rn:R03055  rn:R02269 |
| EC:3.5.2.3 | Dihydroorotase | (S)-Dihydroorotate + H2O <=> N-Carbamoyl-L-aspartate | rn:R01993 |
| EC:3.5.4.1 | Cytosine deaminase | 5-Methylcytosine + H2O <=> Thymine + NH3  Cytosine + H2O <=> Uracil + NH3 | rn:R01411  rn:R00974 |
| EC:3.5.4.12 | dCMP deaminase | dCMP + H2O <=> dUMP + NH3 | rn:R01663 |
| EC:3.5.4.13 | dCTP deaminase | CTP + H2O <=> UTP + NH3  dCTP + H2O <=> dUTP + NH3 | rn:R00568  rn:R02325 |
| EC:3.5.4.30 | dCTP deaminase (dUMP-forming) | dCTP + 2 H2O <=> dUMP + Diphosphate + NH3 | rn:R07307 |
| EC:3.5.4.5 | Cytidine deaminase | Cytidine + H2O <=> Uridine + NH3  Deoxycytidine + H2O <=> Deoxyuridine + NH3 | rn:R01878  rn:R02485 |
| EC:3.6.1.12 | dCTP diphosphatase | dCDP + H2O <=> dCMP + Orthophosphate  dCTP + H2O <=> dCMP + Diphosphate | rn:R01667  rn:R01668 |
| EC:3.6.1.19 | Nucleoside-triphosphate pyrophosphatase | GTP + H2O <=> GMP + Diphosphate  ITP + H2O <=> IMP + Diphosphate  Nucleoside triphosphate + H2O <=> Nucleotide + Diphosphate  UTP + H2O <=> UMP + Diphosphate  XTP + H2O <=> Xanthosine 5'-phosphate + Diphosphate  dGTP + H2O <=> dGMP + Diphosphate  dITP + H2O <=> 2'-Deoxyinosine 5'-phosphate + Diphosphate  dUTP + H2O <=> dUMP + Diphosphate | rn:R00426  rn:R00720  rn:R01532  rn:R00662  rn:R02720  rn:R01855  rn:R03531  rn:R02100 |
| EC:3.6.1.23 | dUTP diphosphatase | dUTP + H2O <=> dUMP + Diphosphate | rn:R02100 |
| EC:3.6.1.5 | Apyrase | ADP + H2O <=> AMP + Orthophosphate  ATP + 2 H2O <=> AMP + 2 Orthophosphate  ATP + H2O <=> ADP + Orthophosphate  CDP + H2O <=> CMP + Orthophosphate  CTP + H2O <=> CDP + Orthophosphate  GDP + H2O <=> GMP + Orthophosphate  GTP + H2O <=> GDP + Orthophosphate  IDP + H2O <=> IMP + Orthophosphate  ITP + H2O <=> IDP + Orthophosphate  Nucleoside triphosphate + H2O <=> NDP + Orthophosphate  UDP + H2O <=> UMP + Orthophosphate  UTP + H2O <=> UDP + Orthophosphate  dTDP + H2O <=> dTMP + Orthophosphate  dTTP + H2O <=> dTDP + Orthophosphate | rn:R00122  rn:R00085  rn:R00086  rn:R00514  rn:R00569  rn:R00328  rn:R00335  rn:R00961  rn:R00719  rn:R02319  rn:R00155  rn:R00159  rn:R02092  rn:R02095 |
| EC:3.6.1.6 | Nucleoside diphosphate phosphatase | GDP + H2O <=> GMP + Orthophosphate  IDP + H2O <=> IMP + Orthophosphate  NDP + H2O <=> Nucleotide + Orthophosphate  UDP + H2O <=> UMP + Orthophosphate | rn:R00328  rn:R00961  rn:R00329  rn:R00155 |
| EC:3.6.1.8 | ATP diphosphatase | ATP + H2O <=> ADP + Orthophosphate  ATP + H2O <=> AMP + Diphosphate  CTP + H2O <=> CMP + Diphosphate  GTP + H2O <=> GMP + Diphosphate  ITP + H2O <=> IMP + Diphosphate  UDP-glucose + H2O <=> UMP + D-Glucose 1-phosphate  UTP + H2O <=> UMP + Diphosphate | rn:R00086  rn:R00087  rn:R00515  rn:R00426  rn:R00720  rn:R00287  rn:R00662 |
| EC:4.1.1.23 | Orotidine-5'-phosphate decarboxylase | Orotidine 5'-phosphate <=> UMP + CO2 | rn:R00965 |
| EC:6.3.4.2 | CTP synthase | ATP + UTP + L-Glutamine + H2O <=> ADP + Orthophosphate + CTP + L-Glutamate  ATP + UTP + NH3 <=> ADP + Orthophosphate + CTP | rn:R00573  rn:R00571 |
| EC:6.3.5.5 | Carbamoyl-phosphate synthase | 2 ATP + HCO3- + NH3 <=> 2 ADP + Orthophosphate + Carbamoyl phosphate  2 ATP + L-Glutamine + HCO3- + H2O <=> 2 ADP + Orthophosphate + L-Glutamate + Carbamoyl phosphate | rn:R07641  rn:R00575 |
